# Loop diuretic therapy with or without heart failure: impact on prognosis

**DOI:** 10.1093/eurheartj/ehae345

**Published:** 2024-06-07

**Authors:** Jocelyn M Friday, John G F Cleland, Pierpaolo Pellicori, Maria K Wolters, John J V McMurray, Pardeep S Jhund, Paul Forsyth, David A McAllister, Fraser J Graham, Yola Jones, Jim Lewsey

**Affiliations:** School of Cardiovascular and Metabolic Health, University of Glasgow, 126 University Place, Glasgow, G12 8TA, UK; School of Cardiovascular and Metabolic Health, University of Glasgow, 126 University Place, Glasgow, G12 8TA, UK; School of Cardiovascular and Metabolic Health, University of Glasgow, 126 University Place, Glasgow, G12 8TA, UK; School of Cardiovascular and Metabolic Health, University of Glasgow, 126 University Place, Glasgow, G12 8TA, UK; Institute for Design Informatics, School of Informatics, University of Edinburgh, Edinburgh, UK; School of Cardiovascular and Metabolic Health, University of Glasgow, 126 University Place, Glasgow, G12 8TA, UK; School of Cardiovascular and Metabolic Health, University of Glasgow, 126 University Place, Glasgow, G12 8TA, UK; Pharmacy Department, NHS Greater Glasgow & Clyde, Glasgow, UK; School of Health and Wellbeing, University of Glasgow, Glasgow, UK; School of Cardiovascular and Metabolic Health, University of Glasgow, 126 University Place, Glasgow, G12 8TA, UK; School of Cardiovascular and Metabolic Health, University of Glasgow, 126 University Place, Glasgow, G12 8TA, UK; School of Health and Wellbeing, University of Glasgow, Glasgow, UK

**Keywords:** Diuretics, Heart failure, Ejection fraction, Left atrium, Epidemiology, Mortality

## Abstract

**Background and Aims:**

Many patients are prescribed loop diuretics without a diagnostic record of heart failure. Little is known about their characteristics and prognosis.

**Methods:**

Glasgow regional health records (2009–16) were obtained for adults with cardiovascular disease or taking loop diuretics. Outcomes were investigated using Cox models with hazard ratios adjusted for age, sex, socioeconomic deprivation, and comorbid disease (adjHR).

**Results:**

Of 198 898 patients (median age 65 years; 55% women), 161 935 (81%) neither took loop diuretics nor had a diagnostic record of heart failure (reference group), 23 963 (12%) were taking loop diuretics but had no heart failure recorded, 7844 (4%) had heart failure recorded and took loop diuretics, and 5156 (3%) had heart failure recorded but were not receiving loop diuretics. Compared to the reference group, five-year mortality was only slightly higher for heart failure in the absence of loop diuretics [22%; adjHR 1.2 (95% CI 1.1–1.3)], substantially higher for those taking loop diuretics with no record of heart failure [40%; adjHR 1.8 (95% CI 1.7–1.8)], and highest for heart failure treated with loop diuretics [52%; adjHR 2.2 (95% CI 2.0–2.2)].

**Conclusions:**

For patients with cardiovascular disease, many are prescribed loop diuretics without a recorded diagnosis of heart failure. Mortality is more strongly associated with loop diuretic use than with a record of heart failure. The diagnosis of heart failure may be often missed, or loop diuretic use is associated with other conditions with a prognosis similar to heart failure, or inappropriate loop diuretic use increases mortality; all might be true.

## Structured Graphical Abstract

**Structured Graphical Abstract:**
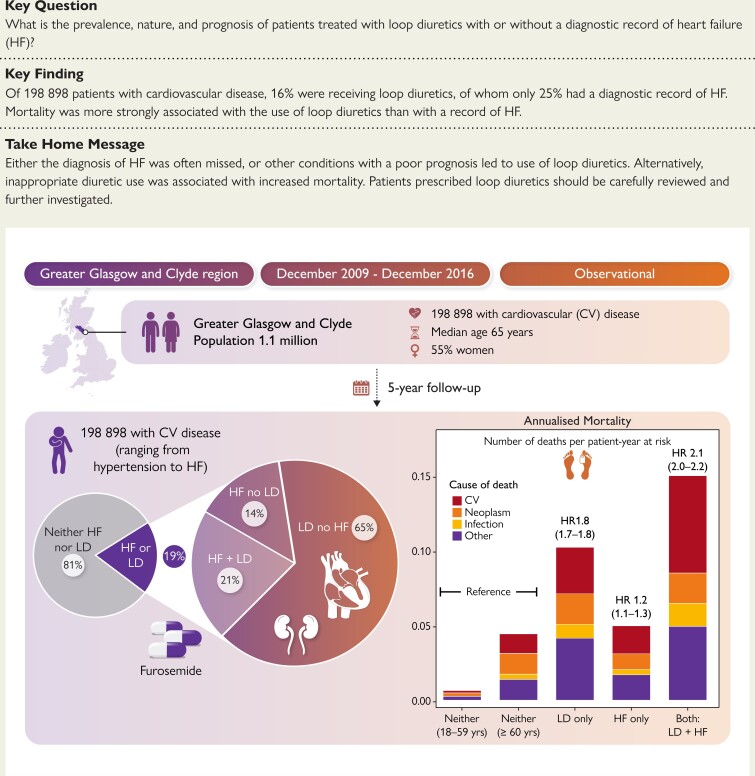

**See the editorial comment for this article ‘Loop diuretics in cardiovascular disease: friend or foe?’, by A. Rosengren, https://doi.org/10.1093/eurheartj/ehae483.**

## Introduction

Heart failure (HF) is characterized by water and salt retention leading to congestion of the systemic and pulmonary circulation and eventually to symptoms, such as exertional breathlessness, and signs, such as peripheral oedema.^1,2^ Guidelines on HF strongly recommend loop diuretics for managing symptoms and signs of congestion,^1, 3–5^ but loop diuretics can also be used for resistant hypertension or managing congestion due to end-stage kidney disease.^1^ For patients with HF, the development of congestion and the need for loop diuretics to manage it are associated with an adverse prognosis.^2^ However, many patients are prescribed loop diuretics but are not investigated to exclude cardiac dysfunction; if they relieve symptoms and signs, they may mask a diagnosis of HF.^1^

To find out how many patients are prescribed loop diuretics, how often this is associated with a diagnosis of HF, and whether prescribing loop diuretics is associated with an adverse prognosis, electronic health records were obtained for patients in the Greater Glasgow & Clyde region who either had a diagnosis of coronary or peripheral arterial disease or HF or were prescribed loop diuretics or medicines commonly prescribed for hypertension or ventricular dysfunction.

## Methods

### Data sources

In Scotland, every resident has free access to primary and secondary healthcare and free prescriptions through the National Health Service (NHS). Residents receive a unique identification number, and all healthcare contacts and deaths are linked through this. For people served by the NHS Greater Glasgow & Clyde Health Board, person-level pseudonymized administrative data were obtained, including demographic data, primary care diagnostic information, hospital admissions and associated diagnostic and procedural codes, community-based prescriptions,^6^ and death records (see Supplementary data online, *Table S3*, for data sources). Any haematology and biochemistry test results from primary and secondary care and reports of electrocardiograms (ECGs) and echocardiograms were also obtained. Data extraction and record linkage were performed by the West of Scotland Safe Haven service. Ethical approval was given by the West of Scotland Safe Haven's Local Privacy Advisory Committee, reference number GSH/18/CA/002.

### Study population

Patients were eligible for inclusion if they were alive and aged ≥18 years on 1 January 2012, had records available for ≥12 months, and had a recorded diagnosis of coronary artery disease (CAD), peripheral arterial disease, or HF between 31 December 2009 and 31 December 2011 or if, for any reason including hypertension, they were dispensed angiotensin-converting enzyme inhibitors (ACEi), angiotensin II receptor blockers (ARB), beta-blockers, mineralocorticoid receptor antagonists (MRA), or loop diuretics (see Supplementary data online, *Tables S1* and *S2*, and *Figure S2*). Diagnoses were identified using either the International Statistical Classification of Diseases and Related Health Problems, 10th Revision (ICD-10) in any position or primary care Read Codes, both with records going back to the year 2000 (see Supplementary data online, *Table S3*). For patients who were included, primary and secondary care diagnostic records were made available from the year 2000 onwards, although prescribing data were not available before 31 December 2009. Medicines dispensed from community pharmacies were identified using British National Formulary codes.

### Patient identification and categorization

Treatment with loop diuretics (see Supplementary data online, *Table S7*, for list) was defined as the first occasion that loop diuretics were dispensed in two consecutive quarters or if death occurred within 90 days of the first prescription. Heart failure was defined as a relevant diagnostic record in either primary care (Read codes) or a hospitalization discharge code in any of six possible diagnostic positions (ICD-10 codes) from the year 2000 onwards. Code lists were developed using previously published research^7^ and through searching for the term ‘heart failure’ in the NHS coding dictionary (see Supplementary data online, *Table S4*). Evidence of structural heart disease was not required, but relevant investigations are reported where available. Following previous conventions,^7^ some patients (*n* = 627) were excluded because of uncertainty about HF diagnosis date (see Supplementary data online, *Table S5* and *Figure S1*). A first diagnosis of HF during a fatal hospitalization (1093 deaths) was not counted as incident HF because of diagnostic uncertainty and lack of information on in-hospital loop diuretic use.

Patients were classified on 31 December 2011 into one of four mutually exclusive groups based on a prior record of HF at any time in the prior 11 years and recurrent dispensing of loop diuretics as (i) neither HF nor on loop diuretic, (ii) loop diuretic only, (iii) HF only, or (iv) HF on loop diuretics. Because the average age of patients in the ‘neither’ group was considerably younger than the other three groups, it was further divided into those aged 18–59 years or ≥60 years.

### Study outcomes

Patients were followed from 1 January 2012 to 31 December 2016. Follow-up was right censored on the date of the last available health record, including blood tests or other investigations or dispensing, to avoid uncertainty about survival status if patients emigrated out of the region as this would otherwise introduce an ‘immortal’ bias. The main outcome of interest was a 5-year all-cause mortality. The primary covariate of interest was group membership, defined by loop diuretic dispensing and HF diagnosis. The 5-year cumulative HF incidence, loop diuretic initiation, cause-specific hospitalization, and mortality were also investigated. Causes of hospitalization were defined by the primary discharge code and mapped onto 12 ICD-10 disease categories. Cause of death was defined from patient records and classified as cardiovascular, neoplastic, infection, other, and unknown (see Supplementary data online, *Table S9*).

### Patient characteristics

Baseline characteristics reported for 1 January 2012 included age, sex, ethnicity, Scottish Index of Multiple Deprivation 2012 quintiles, an area-based measurement of socioeconomic deprivation where lower scores indicate higher levels of deprivation (see online supplementary materials),^8^ comorbidities, medication, blood tests, and, when available, the results of ECGs and echocardiograms.

Comorbidities in the 12 years prior to 1 January 2012 were identified from primary and secondary care records and included hypertension, diabetes mellitus, thyroid disease, atrial fibrillation or flutter (AF), CAD [including myocardial infarction (MI)], valve disease, peripheral arterial disease, stroke, chronic obstructive pulmonary disease (COPD), cancer, liver disease, and dementia. Diagnostic code lists were adapted from CALIBER phenotypes^9^ and previously published research^10^ (see Supplementary data online, *Table S6*). Patients without a diagnostic record for a specific disease were assumed to be free from that condition.

In addition to ACEi, ARB, beta-blockers, MRA, and loop diuretics, information on calcium channel blockers, digoxin, thiazides and thiazide-related diuretics, low-dose aspirin, non-steroidal anti-inflammatory drugs, lipid regulators, bronchodilators, thyroid medications, and hypoglycaemic agents including insulin were reported. Patients were considered to be on these medications if dispensed in the 180 days before 1 January 2012. Medicines were identified by the British National Formulary codes (see Supplementary data online, *Table S7*).

The most recent haemoglobin and estimated glomerular filtration rate (eGFR) measured between 2010 and 2012 were reported. Anaemia was defined as <12.0 g/dL for women and <13.0 g/dL for men,^11^ and eGFR was calculated using the Chronic Kidney Disease-Epidemiology Collaboration equation^12^ without adjusting for ethnicity.^13^

Measurements of left ventricular ejection fraction (LVEF) and left atrial diameter were obtained from routinely available echocardiograms (if available) and heart rhythm and QRS duration from ECGs. Results closest to a diagnosis of HF or initiation of loop diuretics were chosen if there were multiple tests. Left ventricular ejection fraction was considered reduced if <50% and left atrium dilated if >4.0 cm for men and >3.8 cm for women.^14^

### Statistical analyses

Patient characteristics are presented as numbers and percentages for categorical variables and median with interquartile range (IQR) for continuous variables. Numbers and percentages of complete records are displayed for variables with missing data. Percentages of categorical variables refer to complete cases.

Prevalence was estimated within mid-year 2012 regional population estimates,^15^ using sex-stratified, 5-year age bands as the denominator from ages 18 to >90 years.

Admission rates were calculated as the number of admissions per patient-year at risk where the patient was at risk of being admitted (alive, not in hospital, and not lost to follow-up). Allowance was made for the competing risk of death when estimating cumulative initiation of loop diuretics and of incident HF.

A Cox proportional hazard regression model was used to assess between-group differences in all-cause mortality with a robust sandwich-type estimator due to the potential lack of statistical independence between chronic comorbidities.^16^ Due to the large sample size and high statistical power to detect small departures from proportional hazards, the proportional effects were visually checked using log–log plots. The model was adjusted for age, as a continuous, linear value, kidney function using a penalized spline on eGFR with five knots (handling missing eGFR data in online supplementary material, *Figure S11* and *Table S12*), sex, Scottish Index of Multiple Deprivation, and history of common, chronic conditions: hypertension, CAD, peripheral arterial disease, diabetes mellitus, valve disease, AF, stroke, cancer, and dementia were selected based on clinical expertise. Results are reported as hazard ratios (HR) with 95% confidence intervals (CI). The estimated cumulative incidence of death due to cardiovascular disease, infection, neoplasm, or other causes of death and all-cause mortality after initiation of loop diuretics or incident HF were also calculated from sub-distribution estimates.

Time-dependent covariates were used to assess the impact of disease progression on morbidity and mortality. The dates of HF diagnosis and the start of repeated loop diuretic dispensing were used to update loop diuretic/HF group. If both events occurred on the same day, patients were classified as being on the combination to avoid immortal time bias (time-dependent covariate analysis in online supplementary material, *Figure S10*). Crude 5-year morbidity and mortality rates were calculated using person-time at risk, where attributable time was allocated based on time-dependent group status. All-cause mortality was modelled using Cox proportional hazards and time-dependent covariates, using time-dependent comorbidity and loop diuretic/HF group values and baseline age, sex, and Scottish Index of Multiple Deprivation.

Study findings are reported as advised by REporting of studies Conducted using Observational Routinely-collected health Data recommendations.^17^ Data were prepared using Microsoft SQL Server Management Studio (version 17.8.1), and statistical analyses were performed using R (version 4.0.5). Relevant packages are listed in the online supplementary material.

## Results

**Figure 1:**
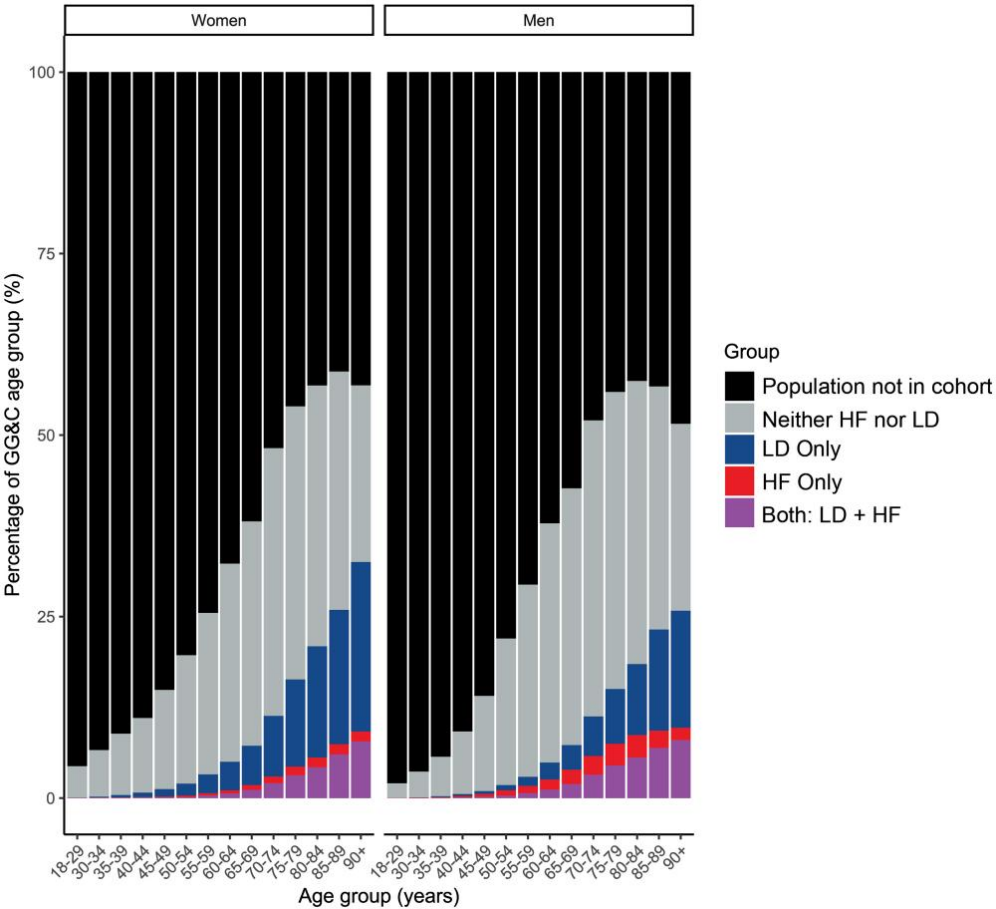
Greater Glasgow & Clyde population classified by sex, age group, repeat prescription of loop diuretics, and a diagnosis of heart failure, based on the mid-year population estimate for 2012. GG&C, Greater Glasgow & Clyde; LD, loop diuretics

Of an estimated 982 385 adults in the NHS Greater Glasgow & Clyde region in 2012, 198 898 met the cohort inclusion criteria, which comprised more than half of all men aged >70 years and more than half of all women aged >75 years (*Figure 1*). The cohort included 161 935 (81%) patients who had no record of HF in the prior decade and had not been repeatedly dispensed loop diuretics (of whom 89 699 were aged ≥60 years), 23 963 (12%) who had been repeatedly dispensed loop diuretics but had no record of HF, 7844 (4%) who had HF and were dispensed loop diuretics, and 5156 (3%) who had a record of HF but were not dispensed loop diuretics. The estimated prevalence of HF for the population (including an estimated 0.3 million people aged <18 years) was 1.0% overall or 1.3% of the adult population (*Figure 1* and *Table S8*).

People aged ≥60 years who neither had a diagnosis of HF nor were taking loop diuretics had a median age of 72 (IQR: 66–78) years, and 54% were women. For these patients, a history of hypertension (38%) and CAD (26%) was common, and 20% had an eGFR < 60 mL/min/1.73 m^2^, 24% had anaemia, and 18% had diabetes mellitus (*Table 1*). Chronic obstructive pulmonary disease (10%), stroke (8%), cancer (8%), AF (7%), and dementia (2%) were less commonly recorded (*Table 1*). Of 77 029 patients who were never subsequently initiated on loop diuretics and did not develop HF, LVEF was <50% in 443 (6%) of 7221 patients with measurements, and the left atrium was dilated in 5884 (46%) of 12 915 patients with measurements (Supplementary data online, *Figures S3*  *and*  *S4*). ECG data are presented in Supplementary data online, *Figure S5*). More than two-thirds of patients were on an ACEi or ARB, 64% on lipid-regulating agents, 45% on beta-blockers, 36% on calcium channel blockers, 35% on thiazides, and 45% on aspirin (*Table 2*). Patients aged 18–59 years who neither had HF recorded nor dispensed loop diuretics had fewer comorbidities than any of the other groups.

**Table 1:** Baseline demographics, comorbidities, and blood tests for the study population classified by prescription of loop diuretics and diagnosis of heart failure

Variable	Neither (18–59 yrs)	Neither (≥60 yrs)	LD only	HF only	Both: LD + HF
*n*	72 236	89 699	23 963	5156	7844
Age (years)	50 (41–55)	72 (66–78)	75 (65–83)	69 (59–78)	77 (68–83)
Under 60 years of age	72 236 (100%)	0 (0%)	3903 (16%)	1358 (26%)	810 (10%)
Sex					
Women	38 838 (54%)	48 184 (54%)	16 775 (70%)	1670 (32%)	3959 (50%)
Men	33 398 (46%)	41 515 (46%)	7188 (30%)	3486 (68%)	3885 (50%)
Ethnicity					
White	44 164 (61%)	70 461 (79%)	20 660 (86%)	4425 (86%)	7151 (91%)
Missing	25 182 (35%)	17 302 (19%)	2902 (12%)	577 (11%)	523 (7%)
Others	2890 (4%)	1936 (2%)	401 (2%)	154 (3%)	170 (2%)
Scottish Index of Multiple Deprivation					
1 (most deprived)	30 476 (42%)	31 527 (35%)	10 525 (44%)	2258 (44%)	3457 (44%)
2	13 046 (18%)	16 252 (18%)	4524 (19%)	917 (18%)	1529 (19%)
3	9857 (14%)	11 914 (13%)	3236 (14%)	613 (12%)	1021 (13%)
4	8170 (11%)	11 904 (13%)	2636 (11%)	582 (11%)	898 (11%)
5 (least deprived)	10 687 (15%)	18 102 (20%)	3042 (13%)	786 (15%)	939 (12%)
HF in hospital records	N/A	N/A	N/A	3702 (72%)	6806 (87%)
Comorbidities^a^					
H/o Hypertension	14 728 (20%)	34 038 (38%)	9110 (38%)	2312 (45%)	4104 (52%)
Diabetes mellitus	8824 (12%)	16 222 (18%)	4858 (20%)	952 (18%)	2303 (29%)
Thyroid disease	709 (1%)	2013 (2%)	1083 (5%)	194 (4%)	502 (6%)
CAD (including MI)	7432 (10%)	23 638 (26%)	7390 (31%)	3860 (75%)	5266 (67%)
MI	3420 (5%)	7916 (9%)	2485 (10%)	2734 (53%)	3157 (40%)
Valve disease	323 (0%)	1338 (1%)	1139 (5%)	507 (10%)	1570 (20%)
Atrial fibrillation/flutter	1021 (1%)	6390 (7%)	3819 (16%)	1284 (23%)	3638 (45%)
Peripheral arterial disease	623 (1%)	2431 (3%)	923 (4%)	291 (6%)	670 (9%)
Stroke	1668 (2%)	7177 (8%)	2385 (10%)	609 (12%)	1183 (15%)
COPD	3321 (5%)	8636 (10%)	4616 (19%)	895 (17%)	2203 (28%)
Liver disease^b^	376 (1%)	293 (0%)	385 (2%)	23 (0%)	64 (1%)
Cancer	1383 (2%)	7308 (8%)	2280 (10%)	417 (8%)	796 (10%)
Dementia	28 (<1%)	1995 (2%)	1195 (5%)	143 (3%)	456 (6%)
Blood results^c^					
Patient record of eGFR	57 305 (79%)	83 815 (93%)	22 575 (94%)	4962 (96%)	7656 (98%)
eGFR	99 (90–106)	78 (64–88)	71 (52–85)	81 (64–92)	61 (43–79)
eGFR 30–59	1061 (2%)	15 469 (18%)	6419 (28%)	886 (18%)	3004 (39%)
eGFR < 30	231 (<1%)	1134 (1%)	1330 (6%)	121 (2%)	707 (9%)
Patient record of haemoglobin	49 507 (69%)	69 406 (77%)	21 125 (88%)	4427 (86%)	7295 (93%)
Haemoglobin: women	13.3 (12.5–14.0)	12.9 (12.0–13.8)	12.6 (11.5–13.6)	12.6 (11.6–13.6)	12.2 (11.1–13.3)
Haemoglobin: men	14.9 (14.0–15.7)	14.1 (13.0–15.1)	13.3 (11.9–14.5)	14.1 (13.0–15.1)	13.2 (11.7–14.4)
Anaemic^d^	5872 (12%)	16 735 (24%)	7696 (36%)	1220 (28%)	3247 (45%)

Data are frequencies (%) for categorical values or median (first–third quartile) for continuous values. Haemoglobin in g/dL.

eGFR, estimated glomerular filtration rate in mL/min/1.73 m^2^ using CKD-EPI equation^12^; H/o hypertension, history of hypertension; CAD, coronary artery disease; MI, myocardial infarction.

^a^History of a coded record on or before 1 January 2012.

^b^Defined by the presence of liver fibrosis, sclerosis, or cirrhosis.

^c^Based on the most recent value in the 2 years before 1 January 2012.

^d^Using the World Health Organization's definition of anaemia.

**Table 2:** Baseline age and concurrent medications for the study population classified by prescription of loop diuretics and diagnosis of heart failure

Variable	Neither (18–59 yrs)	Neither (≥60 yrs)	LD only	HF only	Both: LD + HF
*n*	72 236	89 699	23 963	5156	7844
Age (years)	50 (41–55)	72 (66–78)	75 (65–83)	69 (59–78)	77 (68–83)
Medication^a^					
ACEi or ARB	34 436 (48%)	60 624 (68%)	11 638 (49%)	3992 (77%)	5769 (74%)
ACEi	27 746 (38%)	45 540 (51%)	8498 (35%)	3320 (64%)	4572 (58%)
ARB	7459 (10%)	16 406 (18%)	3687 (15%)	785 (15%)	1465 (19%)
Beta-blocker	27 941 (39%)	40 555 (45%)	8512 (36%)	3542 (69%)	5054 (64%)
MRA	462 (1%)	600 (1%)	971 (4%)	173 (3%)	1179 (15%)
Calcium channel blockers	12901 (18%)	32 213 (36%)	7516 (31%)	1220 (24%)	1737 (22%)
Diltiazem/verapamil	1045 (1%)	3872 (4%)	1801 (8%)	254 (5%)	351 (4%)
Dihydropyridine	11 907 (16%)	28 485 (32%)	5784 (24%)	982 (19%)	1403 (18%)
Digoxin	115 (0%)	1461 (2%)	1792 (7%)	366 (7%)	1772 (23%)
Thiazide^b^	11 151 (15%)	31 103 (35%)	1499 (6%)	591 (11%)	307 (4%)
NSAIDs	12 962 (18%)	11 559 (13%)	3289 (14%)	430 (8%)	441 (6%)
Low-dose aspirin	12 091 (17%)	40 474 (45%)	10 727 (45%)	3383 (66%)	4409 (56%)
Oral anticoagulant	646 (1%)	4114 (5%)	2789 (12%)	722 (14%)	2253 (29%)
Lipid regulator	21 382 (30%)	57 287 (64%)	13 975 (58%)	3999 (78%)	5743 (73%)
Bronchodilators	6691 (9%)	11 580 (13%)	5988 (25%)	839 (16%)	2108 (27%)
Thyroid medications	3375 (5%)	8360 (9%)	3218 (13%)	350 (7%)	949 (12%)
Hypoglycaemic agents	8049 (11%)	13 689 (15%)	4390 (18%)	694 (13%)	1862 (24%)
Insulin^c^	2085 (3%)	2030 (2%)	1196 (5%)	146 (3%)	650 (8%)
Other hypoglycaemic agents	6781 (9%)	12 771 (14%)	3798 (16%)	626 (12%)	1515 (19%)

Data are frequencies (%) for categorical values or median (first–third quartile) for continuous values.

ACEi, angiotensin-converting enzyme inhibitors; ARB, angiotensin receptor blockers; MRA, mineralocorticoid receptor antagonists; NSAIDs, non-steroidal anti-inflammatory drugs.

^a^A prescription dispensed 180 days before through 1 January 2012.

^b^Thiazide or thiazide-related medications.

^c^Either alone or in combination with another agent.

People dispensed loop diuretics but without a record of HF had a median age of 75 (IQR: 65–83) years and were predominantly women (70%), and many (44%) were in the most socioeconomically deprived quintile. Compared with those aged ≥60 years in the ‘neither’ group, they had a similar prevalence of hypertension, diabetes mellitus, CAD, and MI, but more COPD (19%), AF (16%), and anaemia (36%). Although many patients had an eGFR < 60 mL/min/1.73 m^2^ (34%), it was rarely <30 mL/min/1.73 m^2^ (6%). Of the 31 872 patients who received loop diuretics at baseline or follow-up but never received a diagnosis of HF, LVEF was <50% in 509 (9%) of 5409 patients with measurements, and the left atrium was dilated in 5548 (57%) of 9669 patients with measurements. Compared with patients in the ‘neither’ group, patients were less likely to be prescribed ACEi or ARB or thiazide diuretics but more likely to receive bronchodilators and oral anticoagulants. Few patients (15%) received three or more antihypertensive agents (excluding loop diuretics), a criterion commonly used to define resistant hypertension.^18^

People with HF who were not dispensed loop diuretics were younger [median age 69 (IQR: 59–78) years] and predominantly men (68%). Compared with patients taking loop diuretics without a diagnosis of HF, they had a high prevalence of CAD (75%) and MI (53%) and were less likely to have an eGFR < 60 mL/min/1.73 m^2^ (20%). Of 5608 patients who had a diagnosis of HF at baseline or follow-up but who never received loop diuretics, LVEF was <50% in 381 (31%) of 1212 patients with measurements, and the left atrium was dilated in 1112 (55%) of 2022 patients with measurements. Compared with patients taking loop diuretics without a diagnosis of HF, patients with HF only were more likely to receive an ACEi or ARB (77%), beta-blockers (69%), lipid regulators (78%), and aspirin (66%) and less likely to receive bronchodilators (16%).

Patients with a diagnosis of HF who were also treated with loop diuretics had a median age of 77 (IQR 68–83) years, similar to other patients treated with loop diuretics, and 50% were women. Compared with patients taking loop diuretics without a diagnosis of HF, they were more likely to have a history of hypertension (52%), diabetes (29%), CAD (67%), MI (40%), valve disease (20%), AF (45%), COPD (28%), and anaemia (45%). Many patients had an eGFR < 60 mL/min/1.73 m^2^ (48%), but it was rarely <30 mL/min/1.73 m^2^ (9%). Of the 14 938 patients with a diagnosis of HF at baseline or follow-up who also received loop diuretics, LVEF was <50% in 1701 (40%) of 4279 patients with measurements, and the left atrium was dilated in 5545 (74%) of 7518 patients with measurements. Compared with patients taking loop diuretics without a record of HF, they were more likely to receive an ACEi or ARB (74%), beta-blockers (64%), digoxin (23%), and oral anticoagulants (29%) and less likely to receive calcium channel blockers (22%), but only a minority were on MRA (15%).

### Initiation of loop diuretics and incident heart failure during follow-up

**Figure 2:**
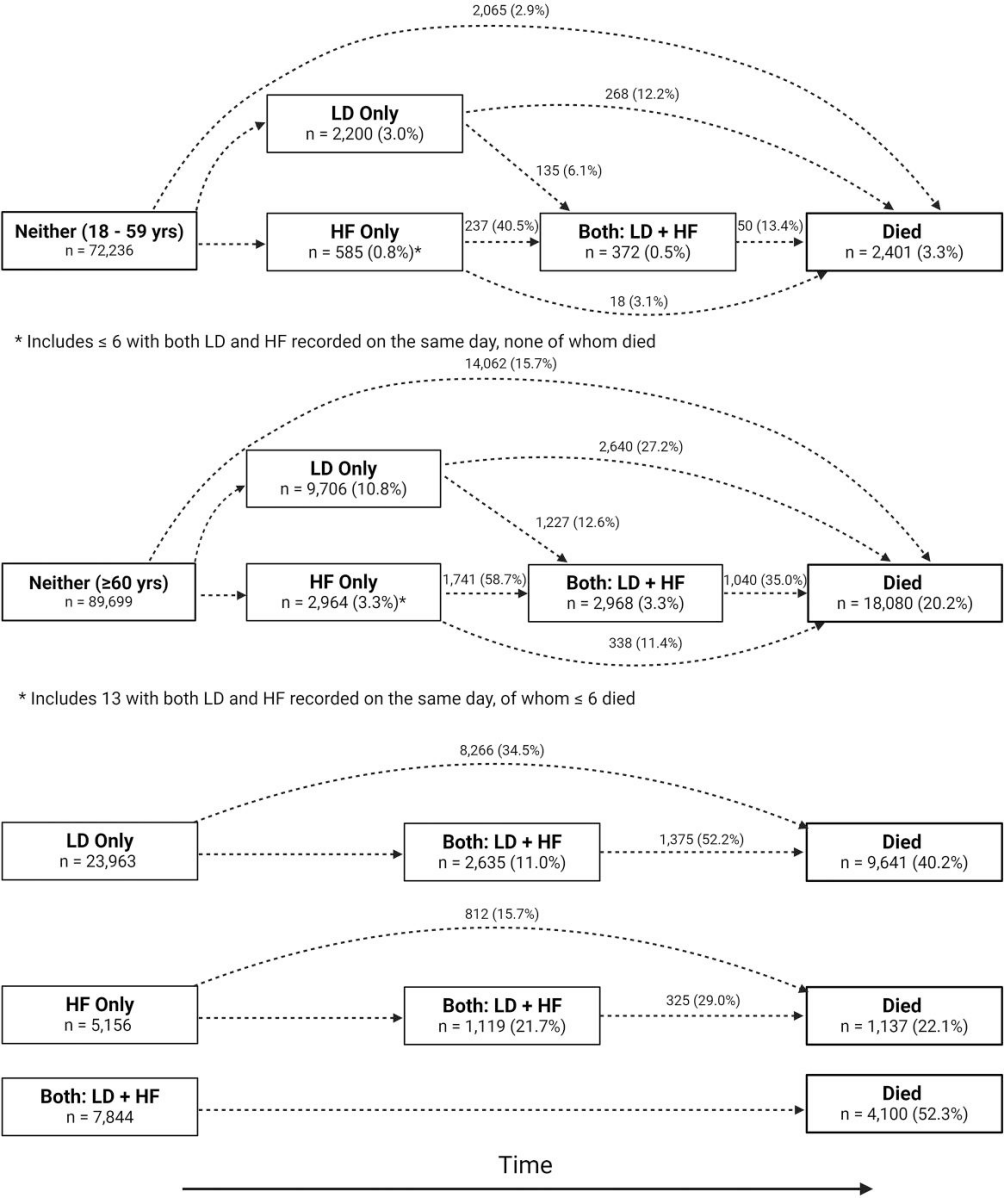
Transition diagrams show how many patients started in each of the four groups (left most boxes) and how many experienced subsequent events between 1 January 2012 and 31 December 2016. Percentages in the boxes are calculated with the baseline group size as the denominator, while transitions are calculated based on those eligible for each transition. Reasons for transitions include diagnosis of heart failure, initiation of loop diuretics, or death during follow-up

**Figure 3:**
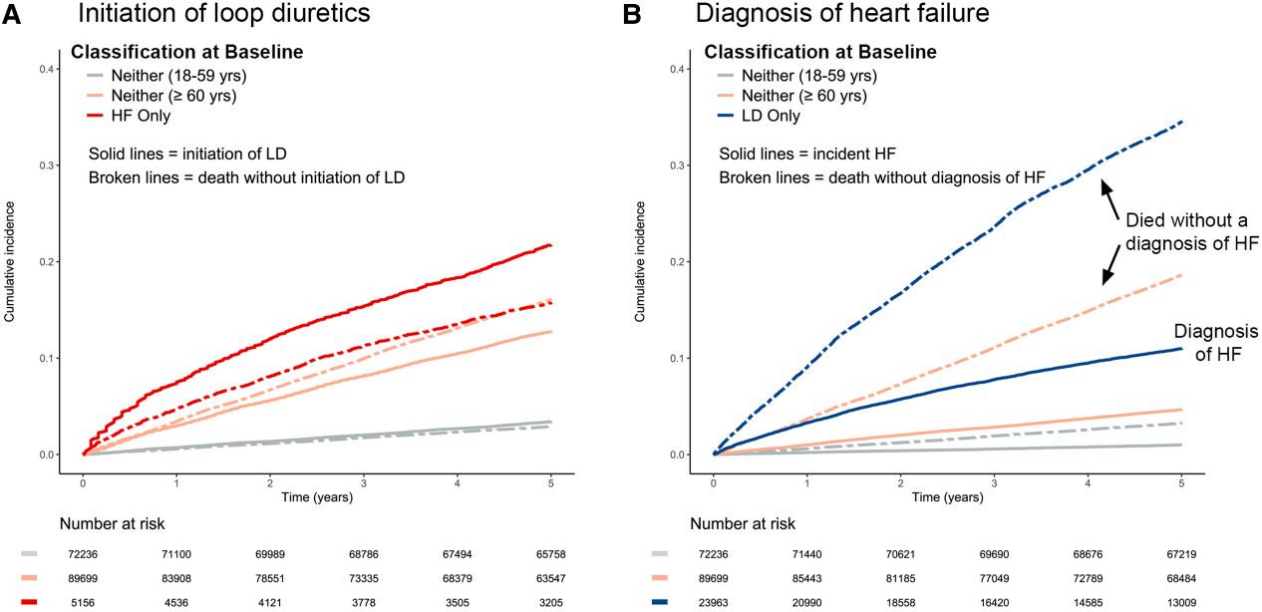
(*A*) Estimation of the cumulative initiation of loop diuretics for patients not already taking loop diuretics at baseline and the competing risk of all-cause mortality. (*B*) Estimation of the cumulative incidence of a diagnosis of heart failure for patients who did not have heart failure at baseline and the competing risk of all-cause mortality. LD, loop diuretics; HF, heart failure; yrs, years

Over the following 5 years, for patients in the ‘neither’ group aged ≥60 years, 9706 (10.8%) started taking loop diuretics, of whom only 12.6% subsequently had HF recorded (*Figure 2*). A further 2951 (3.3%) patients received a diagnosis of HF, of whom 1728 (58.7%) were later initiated on loop diuretics. Overall, 4191 (4.7%) were diagnosed with HF before or after initiation of loop diuretics. For patients initially in the ‘neither’ group and aged ≥60 years (all of whom had evidence of or were receiving treatment for cardiovascular disease), 14 062 (15.7%) died over the following 5 years without receiving a diagnosis of HF or being dispensed loop diuretics. Of those initiated on loop diuretics who did not receive a diagnosis of HF, 2640 (27.2%) subsequently died, and of those initiated on loop diuretics who did receive a diagnosis of HF, 1040 (35.0%) subsequently died (*Figure 3* and Supplementary data online, *Figure S7*). A similar pattern, although at much lower rates, was observed for patients with cardiovascular disease aged 18–59 years (*Figure 2*). Very few deaths (5.7%) occurred within 90 days of first loop diuretic prescription that might have prevented appropriate, timely investigation.

Of 23 963 patients taking loop diuretics at baseline but without a diagnosis of HF, only 2635 (11.0%) subsequently received a HF diagnosis of whom 1375 (52.2%) died; 8266 (34.5%) died without first getting a HF diagnosis (*Figure 2* and *Figure S6*). Of 5156 patients with HF not receiving loop diuretics at baseline, only 1119 (21.7%) were later initiated on loop diuretics of whom 325 (29.0%) died; 812 (15.7%) patients died without being dispensed loop diuretics. For those with HF taking loop diuretics at baseline, mortality was 52.3% (*Figure 2*).

In the overall cohort, 54.4% of deaths were preceded by initiation of loop diuretics or a diagnosis of HF (see Supplementary data online, *Figure S16*). The most common source of new HF diagnosis was hospitalizations. Loop diuretic initiation without a diagnosis of HF generally occurred in primary care (55.0% of initiations) with no secondary care contact (ward or clinic) in the prior 30 days.

### Hospitalizations

**Figure 4:**
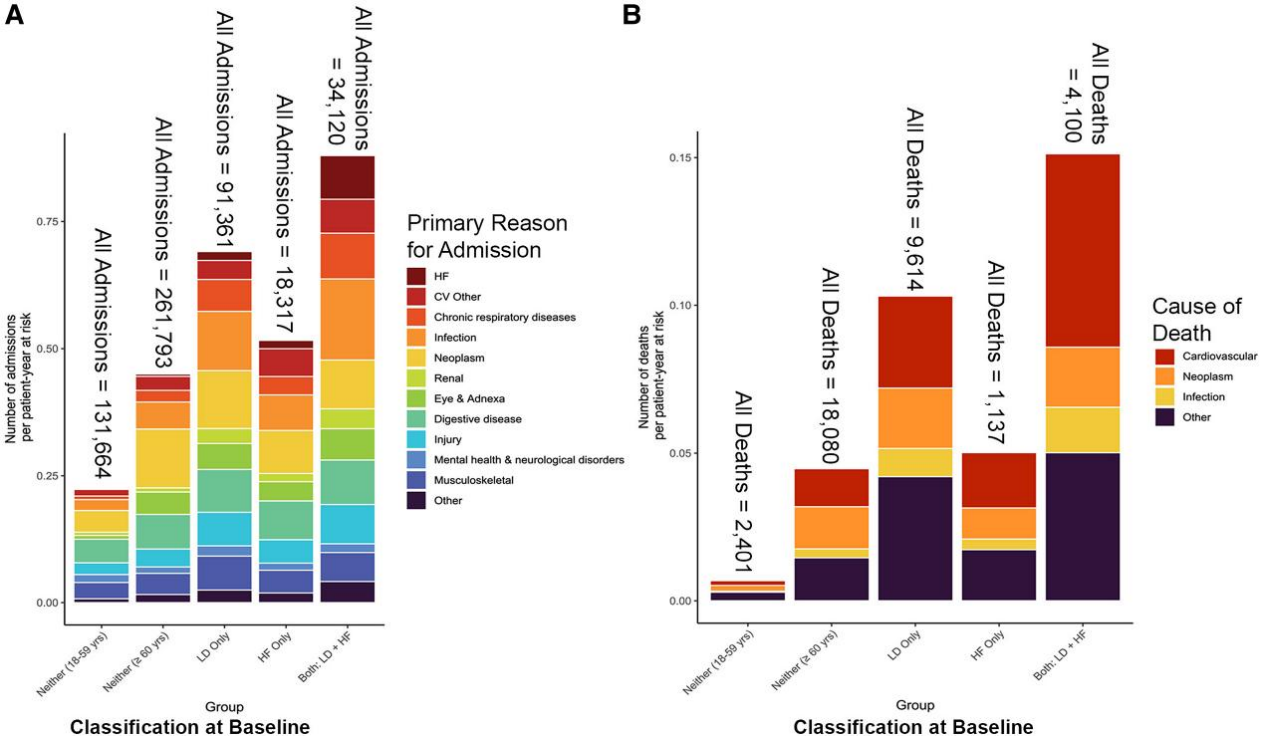
Five-year event rates by patient-year at risk. (*A*) Hospital admission rate classified by the primary admission reason and baseline group determined by the presence or absence of a repeat prescription loop diuretics and a diagnosis of heart failure. Rates were adjusted by patient-year at risk for those eligible (i.e. not already in hospital or dead) to be admitted. The total number of admissions per group is reported above each column. Supplementary data online, *Figures S12*  *and*  *S13*, show similar data with the loop diuretic/HF group as a time-dependent covariate. (*B*) All-cause mortality classified by the underlying cause of death and the baseline group determined by the presence or absence of a repeat prescription of loop diuretic and a diagnosis of heart failure adjusted for patient-year at risk where the patient was under follow-up. Patients were censored at the last medical contact (blood test, prescription, etc.) date to account for patients who moved out of the region. The total number of deaths per group is reported above each column. Supplementary data online, *Figure S1^4^*, shows similar data with loop diuretic/HF group as a time-dependent covariate. *n*, total number of deaths; *N*, total number of admissions

Compared with patients aged ≥60 years who did not have HF and were not taking loop diuretics, rates of hospital admission per patient-year at risk were higher for those taking loop diuretics whether or not they had a diagnosis of HF (*Figure 4*). In the 5 years after 1 January 2012, 39 341 (54.5%) patients in the ‘neither’ group aged 18–59 years had at least 1 admission, with 131 664 admissions in all (0.4 per patient-year at risk), and 66 124 (73.7%) patients in the ‘neither’ group aged ≥60 years had 261 793 admissions (0.7 per patient-year at risk). Patients taking loop diuretics who did not have HF at baseline had 91 361 admissions over 5 years [1.0 per person-year at risk, with 19 884 (83.0%) having at least 1 admission]. Patients with HF who were not dispensed loop diuretic had 18 317 admissions over 5 years [0.8 per person-year at risk, with 4063 (78.8%) having at least 1 admission]. Patients with HF taking loop diuretics had 34 120 admissions over 5 years [1.3 per person-year at risk, with 6842 (87.2%) having at least 1 admission; *Figure 4*]. However, only a minority of admissions for all groups of patients was attributed to cardiovascular disease and an even smaller proportion to HF. Time-dependent analysis showed that rates of admissions increased based on updated HF and loop diuretic status (Supplementary data online, *Figures S12*  *and*  *S13*), especially for cardiovascular and infection-related admissions.

### Mortality

**Figure 5:**
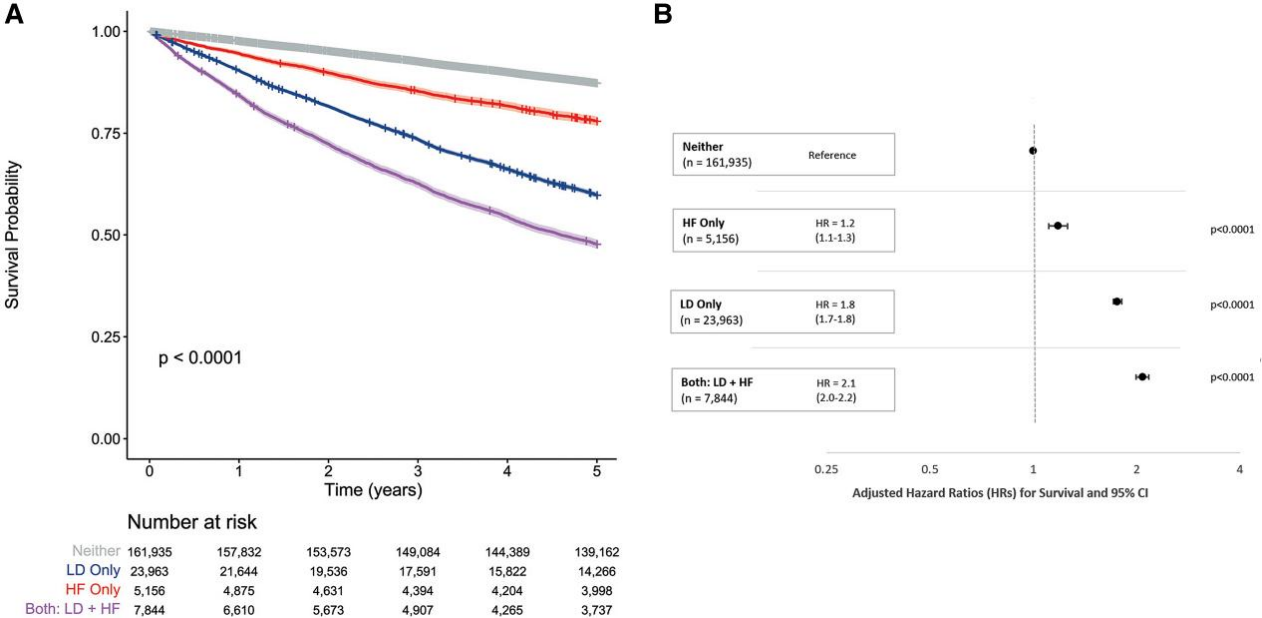
Five-year survival analysis from 1 January 2012 to end of follow-up classified by baseline group according to use of loop diuretics and diagnosis of heart failure. (*A*) Kaplan–Meier curves to compare survival patterns by baseline group. (*B*) Forest plot of hazard ratios with 95% confidence intervals for all-cause mortality by baseline group. The model was adjusted for age, sex, Scottish Index of Multiple Deprivation, a history of hypertension, coronary artery disease, peripheral arterial disease, diabetes mellitus, valve disease, atrial fibrillation or flutter, stroke, cancer, dementia, and the closest eGFR in the prior 2 years

For mortality, the proportional hazard assumption was not met for the first 14 days, but this applied to only 328 deaths (0.9% of all deaths). Accordingly, proportional hazards were considered a reasonable summary of between-group differences. Using the entire ‘neither’ loop diuretics nor HF group as reference, the adjusted HR (which includes age) for 5-year all-cause mortality was 1.8 (95% CI 1.7–1.8) for those dispensed loop diuretics without a diagnosis of HF, 1.2 (95% CI 1.1–1.3) for those with HF who were not dispensed loop diuretics and 2.1 (95% CI 2.0–2.2) for those with HF treated with loop diuretics (*Figure 5*). In the absence of loop diuretics, thiazide diuretics were not associated with adverse outcomes. Applying time-dependent covariates increased the strength of the association between loop diuretics and mortality, especially for patients with HF (see Supplementary data online, *Figure S1^4^*).

All-cause and cardiovascular mortality at 5 years for patients who had neither HF nor were prescribed loop diuretics and were aged <60 years was, respectively, 3.3% and 0.8%; for those ≥60 years, it was 20.2% and 5.8%; for patients with a diagnosis of HF who were not taking loop diuretics, it was 22.1% and 8.2%; for those dispensed loop diuretics without a HF diagnosis, it was 40.2% and 12.1%; and for those with HF taking loop diuretics, it was 52.3% and 22.6% (*Figure 2*; Supplementary data online, *Figures S8*  *and*  *S9*  *and Table S12*). There were similar rates of deaths due to neoplasms for each of these three groups (Supplementary data online, *Figures S8*  *and*  *S15*).

Of 23 963 patients in the loop diuretic-only group, 6506 (79.9% women) were included solely because they were dispensed loop diuretics without meeting other criteria. Of these, 643 had a history of CAD or peripheral arterial disease before 31 December 2009. Of the remaining 5,863, 2251 (38.4%) died. This is similar to the all-cause mortality of 40.2% (7013 deaths) in the remaining 17 457 patients who were included because they also had cardiovascular disease or were prescribed other medications.

## Discussion

This analysis suggests that for patients with a broad range of cardiovascular diseases, mortality is more closely associated with taking loop diuretics than with a diagnosis of HF, even after adjusting for age and other risk factors (*Structured Graphical Abstract*). The estimated prevalence of HF amongst adults in Glasgow of 1.3% is consistent with data from elsewhere in the United Kingdom,^7^ but many more patients (3.2%) were dispensed loop diuretics. Only one in four patients treated with loop diuretics had a diagnostic record of HF, and only 11.0% were subsequently diagnosed with HF over the following 5 years. The prognosis of patients treated with loop diuretics, even without a diagnosis of HF, was substantially worse than that of patients with HF who were not receiving loop diuretics. Indeed, patients with a diagnosis of HF who were not receiving loop diuretics had only a slightly worse prognosis than patients aged ≥60 years with a broad range of other cardiovascular problems. Patients with HF who were treated with loop diuretics had the worst prognosis. Hospitalization rates were also higher for patients taking loop diuretics with or without a diagnosis of HF, although the primary reason was usually for conditions other than cardiovascular disease for all patient groups. In summary, patients dispensed loop diuretics constitute a much larger healthcare problem than patients with a diagnostic record of HF, which might be explained by substantial under-diagnosis or under-recording of HF.^1^ This has serious implications for HF epidemiology and health service capacity to manage it. Patients with cardiovascular disease treated with loop diuretics are at high risk even if they do have no record of HF, which should alert clinicians to consider the need for further investigation and treatment.

The observation that loop diuretics are associated with an adverse prognosis in the absence of HF is not unique to Glasgow. International trials of AF^19^ and type 2 diabetes mellitus^20^ show that patients treated with loop diuretics often do not carry a diagnosis of HF but have worse outcomes than those with a diagnostic label of HF who are not treated with loop diuretics; those with both HF and loop diuretics consistently have the worst outcome. A study of patients with AF from England found that those receiving loop diuretics but no record of HF had a prognosis similar to those with HF recorded.^21^

Current criteria used to define HF are not robust, relying heavily on symptoms and signs such as breathlessness and ankle swelling, that lack specificity and may not be obvious until HF is severe, leading to hospitalization.^1,7,22–24^ In the current analysis, few patients initiated on loop diuretics without a diagnosis of HF had measured natriuretic peptides or an echocardiogram to support or exclude a diagnosis of HF. However, many patients had hypertension, diabetes mellitus, anaemia, AF, and impaired kidney function, common comorbidities that may cause or exacerbate HF. The diagnosis of HF in the presence of a preserved LVEF is especially difficult when other reasons for symptoms and signs exist, such as lung or kidney disease.^25^ Many patients treated with loop diuretics who were not given a diagnosis of HF were older women and had left atrial dilation, consistent with the demographic profile and diagnosis of HF with preserved ejection fraction. Atrial fibrillation may also cause left atrial dilation, but if it leads to symptoms and signs of congestion requiring treatment with loop diuretics, it may also be considered to have caused HF. Such diagnostic uncertainties might explain why many patients are treated with loop diuretics but are not diagnosed with HF.

About half of patients had been hospitalized in the year before initiation of loop diuretics, and many others attended hospital outpatients, usually for non-cardiovascular conditions. A few patients treated with loop diuretics had end-stage kidney disease, and some will have received loop diuretics for the treatment of resistant hypertension, but neither indication for loop diuretics appeared to account for their widespread use.^3^ On general medical and surgical wards, loop diuretics may have been initiated for symptoms and signs of HF, but appropriate investigations were not done or not recorded in patients’ medical records.^26^ When loop diuretics are listed as hospital discharge medications, primary care physicians may automatically repeat the prescription, trusting that their hospital colleagues have investigated appropriately. Patients admitted with, e.g. respiratory infections, may receive both antibiotics and loop diuretics due to diagnostic uncertainty. Even if antibiotics are responsible for the success of treatment, loop diuretics may be continued long term because no one decided to stop them.

Current consensus guidelines state that the symptoms and signs of congestion are essential diagnostic criteria for HF^22^ and strongly recommend loop diuretics for their treatment.^4^ In the current analysis, many patients with a diagnostic label of HF were not treated with loop diuretics. These patients had often had MIs, had reduced LVEF, and were prescribed renin–angiotensin system inhibitors and beta-blockers. The relatively favourable prognosis of patients with HF who were not treated with loop diuretics may reflect the effective deployment of guideline-recommended therapies that prevent or reverse the development of congestion, rendering treatment with loop diuretics unnecessary. However, the decision not to initiate loop diuretics suggests that these patients had few or no symptoms or signs of congestion and may not have fulfilled current guideline criteria for a diagnosis of HF.

That there is an association between taking loop diuretics and an adverse prognosis in patients with HF is not surprising.^27^ Loop diuretics are used to treat symptoms and signs of congestion, which is associated with more severe cardiac and renal dysfunction and a higher mortality. For patients with severe congestion, loop diuretics are almost certainly life-saving in the short term, but the longer-term adverse consequences, including activation of neuroendocrine systems, electrolyte disturbances, and increased calcium excretion, might increase morbidity and mortality.^28–30^ The overall prognosis of patients taking loop diuretics will reflect the average prognosis for each of the diverse reasons for their use. Patients who received loop diuretics for ankle swelling in the absence of serious underlying disease might have a good prognosis, and therefore, for other patients within this diverse group, it must be much worse. It is also possible that prognosis is driven primarily by problems other than HF, such as lung disease or cancer, for which loop diuretics might do more harm than good. Further research is required to determine how often loop diuretic prescription is simply a bystanding marker of a poor outcome or associated with symptoms and signs due to cardiac dysfunction—in other words, HF.

### Strengths and limitations

Administrative health records were accessed for a region encompassing 23% of the Scottish population,^15^ including all community-based prescriptions and blood tests from 2010 onwards and primary care diagnosis and reasons for hospital admissions from the year 2000 onwards. However, some important variables were unavailable, including height, weight, smoking habit, blood pressure, doses of medicines and their frequency. Unlike many other large administrative datasets,^9,31,32^ a large sample of echocardiographic and ECG results were available. However, many more tests may have been done at the ‘bedside’ without results being entered into electronic records. In future, accessing patients’ case notes may be possible to retrieve this information. Information on in-hospital prescribing of interventions such as intravenous loop diuretic use was not available.

The cohort was defined with a broad, though incomplete, set of cardiovascular diseases, missing those with conditions such as AF, valvular disease, or venous thromboembolism. However, any patient dispensed an ACEi, ARB, MRA, beta-blocker, or loop diuretic would still have been included.

Hypertension was probably under-reported, given the high prescription rates for ACEi, ARB, calcium channel blockers, and thiazide diuretics. However, the prevalence of diabetes and percentage of patients on hypoglycaemic therapy and the prevalence of COPD and percentage on bronchodilators were similar, suggesting that these are useful pharmaco-epidemiological markers of disease,^33,34^ just as loop diuretics might be for symptoms and signs of HF. Research using administrative health records is reliant on clinical coding. In an audit of Scottish hospitalization records, the ‘I50’ code for HF was judged to be correct >90% of the time but missed a diagnosis of HF in 22% of cases.^35^ Death certificates are probably fairly accurate for classifying deaths as cardiovascular or due to cancer but may be less reliable for further specifying causes such as HF or sudden death.

In conclusion, this analysis suggests that in patients with a broad range of cardiovascular diseases, mortality is more strongly associated with use of loop diuretics than with a diagnosis of HF, although amongst those treated with loop diuretics, a diagnosis of HF is associated with a worse prognosis. Either the diagnosis of HF is often missed, which may result in the withholding of evidence-based treatment for HF, or loop diuretic use is associated with other conditions with a prognosis similar to HF, or inappropriate loop diuretic use increases mortality; all might be true and contribute to poor outcomes. Nevertheless, treatment with loop diuretics provides a simple, reliably collected, objective marker of patients at an increased risk of hospitalization and death. The proportion of patients on loop diuretics undergoing investigation for HF could be used to audit the quality of diagnostic care in clinical practice. When a clinician encounters a patient treated with loop diuretics, this should trigger a review of the patient's medical records to ensure that appropriate investigations have been done, such as measurement of natriuretic peptides or cardiac imaging, to exclude serious cardiac pathology.

## Supplementary Material

ehae345_Supplementary_Data

## Declarations

### Data Availability

The data and study materials will not be made available to other researchers. However, the analysis code is available on GitHub (https://github.com/jocelynfriday/CharactersticsAndPrognosisOfLD), individual-level datasets can be requested for use in other research studies through an application to NHS West of Scotland Safe Haven (https://www.researchdata.scot/safe-haven-services), and aggregated data are provided in the supplementary material.

### Pre-registered Clinical Trial Number

None supplied.

## References

[1] Cleland JGF, Pfeffer MA, Clark AL, Januzzi JL, McMurray JJV, Mueller C, et al The struggle towards a universal definition of heart failure—how to proceed? Eur Heart J 2021;42:2331–43. 10.1093/eurheartj/ehab082 33791787

[2] Pellicori P, Cleland JG, Zhang J, Kallvikbacka-Bennett A, Urbinati A, Shah P, et al Cardiac dysfunction, congestion and loop diuretics: their relationship to prognosis in heart failure. Cardiovasc Drugs Ther 2016;30:599–609. 10.1007/s10557-016-6697-7 27819111

[3] National Institute for Health and Care Excellence . British National Formula—BNF Diuretics Treatment Summary NICE; 2018. https://bnf.nice.org.uk/treatment-summary/diuretics.html (20 January 2019).

[4] McDonagh TA, Metra M, Adamo M, Gardner RS, Baumbach A, Böhm M, et al 2021 ESC Guidelines for the diagnosis and treatment of acute and chronic heart failure: developed by the task force for the diagnosis and treatment of acute and chronic heart failure of the European Society of Cardiology (ESC) with the special contribution of the Heart Failure Association (HFA) of the ESC. Eur Heart J 2021;42:3599–726. 10.1093/eurheartj/ehab368 34447992

[5] Mebazaa A, Solal AC, Colombo PC. Assessing and treating congestion in acute decompensated heart failure: are we seeing the light at the end of the tunnel? European heart journal 2023;44:51–104 1. 36426405 10.1093/eurheartj/ehac680

[6] Alvarez-Madrazo S, McTaggart S, Nangle C, Nicholson E, Bennie M. Data resource profile: the Scottish National Prescribing Information System (PIS). Int J Epidemiol 2016;45:714–5f. 10.1093/ije/dyw060 27165758 PMC5005947

[7] Conrad N, Judge A, Tran J, Mohseni H, Hedgecott D, Crespillo AP, et al Temporal trends and patterns in heart failure incidence: a population-based study of 4 million individuals. Lancet 2018;391:572–80. 10.1016/S0140-6736(17)32520-5 29174292 PMC5814791

[8] The Scottish Government . Scottish Index of Multiple Deprivation 2012. Edinburgh: National Statistics, 2012.

[9] Denaxas SC, George J, Herrett E, Shah AD, Kalra D, Hingorani AD, et al Data resource profile: cardiovascular disease research using linked bespoke studies and electronic health records (CALIBER). Int J Epidemiol 2012;41:1625–38. 10.1093/ije/dys188 23220717 PMC3535749

[10] Reeves D, Springate DA, Ashcroft DM, Ryan R, Doran T, Morris R, et al Can analyses of electronic patient records be independently and externally validated? The effect of statins on the mortality of patients with ischaemic heart disease: a cohort study with nested case–control analysis. BMJ Open 2014;4:e004952. 10.1136/bmjopen-2014-004952 PMC4010839 24760353

[11] World Health Organization . Haemoglobin concentrations for the diagnosis of anaemia and assessment of severity. World Health Organization, 2011.

[12] Levey AS, Stevens LA, Schmid CH, Zhang YL, Castro AF 3rd, Feldman HI, et al A new equation to estimate glomerular filtration rate. Ann Intern Med 2009;150:604–12. 10.7326/0003-4819-150-9-200905050-00006 19414839 PMC2763564

[13] Delgado C, Baweja M, Crews DC, Eneanya ND, Gadegbeku CA, Inker LA, et al A unifying approach for GFR estimation: recommendations of the NKF-ASN task force on reassessing the inclusion of race in diagnosing kidney disease. Am J Kidney Dis 2022;79:268–88.e1. 10.1053/j.ajkd.2021.08.003 34563581

[14] Lang RM, Badano LP, Mor-Avi V, Afilalo J, Armstrong A, Ernande L, et al Recommendations for cardiac chamber quantification by echocardiography in adults: an update from the American Society of Echocardiography and the European Association of Cardiovascular Imaging. Eur Heart J Cardiovasc Imaging 2015;16:233–71. 10.1093/ehjci/jev014 25712077

[15] National Records of Scotland, Mid-year population estimates by pre-April 2014 NHS Board areas by single year of age and sex: 1981-2013, 2018. https://www.nrscotland.gov.uk/files//statistics/time-series/population/hbe8113-pre-14-nhs-board-areas-revised.xlsx. (27 June 2022).

[16] Therneau TM . A package for survival analysis in R. R package version 3.4-0 ed, 2022. https://cran.r-project.org/src/contrib/Archive/survival/.

[17] Benchimol EI, Smeeth L, Guttmann A, Harron K, Moher D, Petersen I, et al The REporting of studies Conducted using Observational Routinely-collected health Data (RECORD) statement. PLoS Med 2015;12:e1001885. 10.1371/journal.pmed.1001885 26440803 PMC4595218

[18] Carey RM, Calhoun DA, Bakris GL, Brook RD, Daugherty SL, Dennison-Himmelfarb CR, et al Resistant hypertension: detection, evaluation, and management: a scientific statement from the American Heart Association. Hypertension 2018;72:e53–90. 10.1161/HYP.0000000000000084 30354828 PMC6530990

[19] Cleland JGF, Shelton R, Nikitin N, Ford S, Frison L, Grind M. Prevalence of markers of heart failure in patients with atrial fibrillation and the effects of ximelagatran compared to warfarin on the incidence of morbid and fatal events: a report from the SPORTIF III and V trials. Eur J Heart Fail 2007;9:730–9. 10.1016/j.ejheart.2007.01.013 17360232

[20] Pellicori P, Fitchett D, Kosiborod MN, Ofstad AP, Seman L, Zinman B, et al Use of diuretics and outcomes in patients with type 2 diabetes: findings from the EMPA-REG OUTCOME trial. Eur J Heart Fail 2021;23:1085–93. 10.1002/ejhf.2220 34031968 PMC11497224

[21] Zakeri R, Morgan AD, Sundaram V, Bloom C, Cleland JGF, Quint JK. Under-recognition of heart failure in patients with atrial fibrillation and the impact of gender: a UK population-based cohort study. BMC Med 2021;19:179. 10.1186/s12916-021-02048-8 34372832 PMC8353868

[22] Bozkurt B, Coats AJS, Tsutsui H, Abdelhamid CM, Adamopoulos S, Albert N, et al Universal definition and classification of heart failure: a report of the Heart Failure Society of America, Heart Failure Association of the European Society of Cardiology, Japanese Heart Failure Society and Writing Committee of the Universal Definition of Heart Failure. Eur J Heart Fail 2021;23:352–80. 10.1002/ejhf.2115 33605000

[23] Mosterd A, Hoes AW. Clinical epidemiology of heart failure. Heart 2007;93:1137–46. 10.1136/hrt.2003.025270 17699180 PMC1955040

[24] Shah SJ, Katz DH, Deo RC. Phenotypic spectrum of heart failure with preserved ejection fraction. Heart Fail Clin 2014;10:407–18. 10.1016/j.hfc.2014.04.008 24975905 PMC4076705

[25] Lam CSP, Solomon SD. Classification of heart failure according to ejection fraction: JACC review topic of the week. J Am Coll Cardiol 2021;77:3217–25. 10.1016/j.jacc.2021.04.070 34167646

[26] Cleland JG, McDonagh T, Rigby AS, Yassin A, Whittaker T, Dargie HJ, et al The national heart failure audit for England and Wales 2008–2009. Heart 2011;97:876–86. 10.1136/hrt.2010.209171 21173198

[27] Damman K, Kjekshus J, Wikstrand J, Cleland JG, Komajda M, Wedel H, et al Loop diuretics, renal function and clinical outcome in patients with heart failure and reduced ejection fraction. Eur J Heart Fail 2016;18:328–36. 10.1002/ejhf.462 26693947

[28] Lim LS, Fink HA, Blackwell T, Taylor BC, Ensrud KE. Loop diuretic use and rates of hip bone loss and risk of falls and fractures in older women. J Am Geriatr Soc 2009;57:855–62. 10.1111/j.1532-5415.2009.02195.x 19368583 PMC2721719

[29] Bruderer S, Bodmer M, Jick SS, Meier CR. Use of diuretics and risk of incident gout: a population-based case–control study. Arthritis Rheumatol 2014;66:185–96. 10.1002/art.38203 24449584

[30] Flamenbaum W . Diuretic use in the elderly: potential for diuretic-induced hypokalemia. Am J Cardiol 1986;57:A38–43. 10.1016/0002-9149(86)91005-2 3511656

[31] Public Health Scotland . ISD Datamarts 2020. https://www.isdscotland.org/Products-and-Services/Datamarts/ISD-Datamarts/ (9 September 2022).

[32] Jones KH, Ford DV, Thompson S, Lyons R. A profile of the Sail Databank on the UK secure research platform. Int J Popul Data Sci 2019;4:1134. 10.23889/ijpds.v4i2.1134 34095541 PMC8142954

[33] Takahashi Y, Nishida Y, Asai S. Utilization of health care databases for pharmacoepidemiology. Eur J Clin Pharmacol 2012;68:123–9. 10.1007/s00228-011-1088-2 21808989

[34] Furu K, Wettermark B, Andersen M, Martikainen JE, Almarsdottir AB, Sørensen HT. The Nordic countries as a cohort for pharmacoepidemiological research. Basic Clin Pharmacol Toxicol 2010;106:86–94. 10.1111/j.1742-7843.2009.00494.x 19961477

[35] Data Quality Assurance . Assessment of SMR01 Data Scotland 2014–2015, 2019. https://www.isdscotland.org/Products-and-Services/Data-Quality/docs/Assessment-of-SMR01-Data-2014-15-report-181019.pdf (24 August 2022).

