# Supplementary material for: Loop diuretic therapy with or without heart failure: impact on prognosis
Source: Eur Heart J. 2024 Jun 7;45(37):3837–49. doi: 10.1093/eurheartj/ehae345 (PMC11452746; doi:10.1093/eurheartj/ehae345)
Supplement: ehae345_Supplementary_Data [file ehae345_supplementary_data.pdf]

Table of Contents

Coding Systems 3

Cohort definition 3

Supplementary Table 1 Database and codes used to generate the cohort. 4

Supplementary Table 2 Codes used to identify a history of CAD, PAD, HF, or a prescription for an ACEi, ARB, MRA, beta-blocker, or loop diuretics. 5

Supplementary Figure 1 Flow chart of inclusion and exclusion of NHS Greater Glasgow & Clyde patients 12

Supplementary Figure 2 Venn diagram of patients' status on cohort inclusion on 31 December 2011 13

Data sources and coverage 13

Supplementary Table 3 Notes on data sources used for analysis, including date ranges and coverage.13

Classification of heart failure, medications, socioeconomic deprivation, and co-morbidities 15

Supplementary Table 4 ICD-10 and NHS Greater Glasgow & Clyde formatted Read Codes used to identify patients with heart failure. 15

Supplementary Table 5 NHS Greater Glasgow & Clyde formatted Read Codes used to exclude heart failure cases where the code refers to an existing diagnosis after 31 December 2011. 17

Supplementary Table 6 Description of how co-morbidities were defined and the data sources used to define them. 18

Supplementary Table 7 Prescribed medication classifications, categories each medication was assigned to, the portion of the BNF code used to select for the category, and the description of the BNF selection code. 18

Scottish Index of Multiple Deprivation 20

R packages 20

Aggregated data 20

Supplementary Table 8 Cohort group by age and sex against the estimated mid-year NHS Greater Glasgow & Clyde Health Board's mid-year 2012 population estimate. 20

Supplementary Figure 3 Left ventricular ejection fraction (LVEF) data classified by prescription of loop diuretics (LD) and a diagnosis of heart failure (HF) 23

Supplementary Figure 4 Left atrial diameter data classified by prescription of loop diuretics (LD) and a diagnosis of heart failure (HF) 24

Supplementary Figure 5 Heart rhythm on 12-lead ECG 25

Classification of morbidity and mortality events 26

Supplementary Table 9 Definitions of disease categories used for classifying causes of death and hospitalisations. 26

Supplementary Table 10 Codes used to define infection admissions in SMR01 27

Supplementary Table 11 Codes used to define chronic respiratory disease admissions in SMR01 28

Supplementary Figure 6 5-year cumulative incidence of loop diuretic (LD) initiation or diagnosis of heart failure (HF) 29

Supplementary Figure 7: 4-year mortality from initiation of loop diuretics (LD) or diagnosis of heart failure (HF) during follow-up 30

Supplementary Figure 8 5-year cumulative incidence of causes of death by baseline group classification on 31 December 2011. 32

Data on the loop diuretic only population 33

Supplementary Table 12 Demographics and co-morbidities of individuals with loop diuretics only at baseline classified by survival status at the end of follow-up 33

Supplementary Figure 9 5-year Kaplan-Meier curves to compare survival rates of those with only a loop diuretic at baseline by quintile of age as of 1 January 2012. 34

Time-dependent covariate analysis 34

Supplementary Figure 10 Explanation of changes in assigning patient time at risk based on the analysis method. 35

Handling missing eGFR data 35

Supplementary Figure 11 Changes in 5-year all-cause mortality adjusted hazard ratios based on using complete eGFR case analysis against replacing missing values in turn with available per group median value, first quartile per group value, and third quartile per group value. 36

Supplementary Table 12 Median (1st-3rd quartile) values of eGFR at baseline and time-dependent status for the study population classified by prescription of loop diuretics (LD) and diagnosis of heart failure (HF) 37

Supplementary Figure 12 Number and type of admissions per person-year at risk from 1 January 2012 through 31 December 2016, where the group is a time-dependent covariate. 38

Supplementary Figure 13 Admission rates using baseline versus time-dependent classifications. 39

Supplementary Figure 14 5-year mortality using time-dependent groups. 40

Supplementary Figure 15 Mortality rates using baseline versus time-dependent classifications. 41

Supplementary Figure 16: Classification of group status at time of death using (A) baseline and (B) time-dependent classification prior to death during 5 years of follow-up. 42

References 42

Coding Systems

The analysis used four coding schemes to identify the presence, and assumed absence, of cohort inclusion factors, comorbidities, and medications.

The International Statistical Classification of Diseases and Related Health Problems, 10th Revision (ICD-10), is a hierarchical medical classification system and the global standard for morbidity and mortality statistics provided by the World Health Organization (WHO). Unless explicitly noted otherwise, three- and four-digit ICD-10 codes also include all codes below them.

The Office of Population Census and Surveys Classification of Interventions and Procedures, Version 4 (OPCS-4) coding standard is maintained, licensed, and supported by the NHS Digital Terminology and Classification Delivery Service and the standard is governed by Crown Copyright (1). OPCS-4 is a hierarchical coding standard used to classify operations, procedures, and interventions conducted within the NHS. For Scotland, Public Health Scotland Terminology Services provides help and support to NHS Scotland with implementing the Scottish Clinical Coding Standards.

The British National Formulary (BNF) is a joint reference book published by the British Medical Association and Royal Pharmaceutical Society. It contains the standard list of medications used within the NHS. Unique, hierarchical codes are assigned to medications provided on the NHS. The first nine characters specify the chemical level of the medication.

Read Codes are a hierarchical controlled clinical vocabulary of terms and short phrases used for British primary care data. The original version of Read Codes used a standardised 4-byte set, which version 2 extended to a 5-byte unified set. The 5-byte version 2 code added a term code to hold an 'idea' or 'concept'; where the preferred term had '00' appended, and synonyms append term codes 11-99 (2). For example, if the original 5-byte Read Code was 'G630..' for myocardial infarction, the 5-byte version 2 code, with the preferred term code, is 'G630..00' and the first synonym, heart attack, is 'G30..11', followed by coronary thrombosis, 'G30..12'. The NHS Greater Glasgow & Clyde GP data used the 5-byte set of codes without the term code, meaning synonyms are mapped onto the same 5-digit code. For example, 'G580.11', Congestive cardiac failure, and 'G580.00', Congestive heart failure' both map onto 'G580.'. Additionally, trailing space holders (.) have been removed, so 'G580.11' maps to 'G580' and 'G58..00' maps to 'G58'. Clinicians are asked to code the clinical information as specifically as possible, and nurses are asked to code an activity and the underlying disease or condition that led to the activity being carried out (3).

Cohort definition

This analysis used an existing cohort. The cohort was defined out of the subset of patients receiving care from the NHS Greater Glasgow & Clyde Health Board between 31st December 2009 through cohort extraction in 2018 with a record of at least one of the codes listed below between 31 December 2009 through 31 December 2011 in Supplementary Table 1. For the purposes of this analysis, we used an expanded list of codes to follow the president set out by previous studies (see Supplementary Table 2). We felt justified in making this decision because a cohort generated from the codes in Supplementary Table 2 only captures 6,872 individuals eligible to be included. This represents an increase of 1.7% of the individuals who would be potentially eligible for inclusion before applying inclusion and exclusion criteria.

Supplementary Table 1 Database and codes used to generate the cohort.

Patients were included based on the existence of a record across at least one of community-based prescriptions, primary care, hospital admission records, or mental health inpatient and day case records between 31 December 2009 through 31 March 2018.

Codes are listed based on the formatting used within Safe Haven, and unless explicitly noted otherwise, three- and four-digit ICD-10 codes include all codes below them. BNF codes include all codes below them.

Dataset	Code Type	Variable	Code	Description
SMR01/04	ICD-10	PAD	I73	Other peripheral vascular disease
SMR01/04	ICD-10	CAD	I24	Other Acute ischaemic heart disease
SMR01/04	ICD-10	CAD	I254	Coronary artery aneurism and dissection
SMR01/04	ICD-10	CAD	Z951	Presence of aortocoronary bypass graft
SMR01/04	ICD-10	CAD	Z955	Presence of coronary angioplasty implant and graft
SMR01/04	ICD-10	HF	I50	Heart failure
SMR01/04	ICD-10	HF	I110	Hypertensive heart disease with (congestive) heart failure
SMR01/04	ICD-10	HF	I130	Hypertensive heart and renal disease with (congestive) heart failure
GP LES	Read	PAD	G734	Echocardiogram shows left ventricular systolic dysfunction
GP LES	Read	CAD	G340	Coronary artery disease (CAD)
GP LES	Read	HF	G5yy9	Left ventricular systolic dysfunction (LVSD)
GP LES	Read	HF	585f	Echocardiogram shows left ventricular systolic dysfunction
GP LES	LES Area	HF	4	Heart failure
PIS	BNF	ACEi	0205051	Angiotensin-converting-enzyme inhibitor (ACEi)
PIS	BNF	ARB	0205025	Angiotensin II receptor blocker (ARB)
PIS	BNF	Beta-blocker	0204	Beta-blocker
PIS	BNF	LD	020202	Loop diuretics (LD)
PIS	BNF	MRA	020203	Mineralocorticoid receptor antagonists (MRA)

SMR01, Scottish Morbidity Records for Acute Inpatient and Day Cases; SMR04, Mental Health Inpatient and Day Cases; GP LES, General Practice Local Enhanced Services; PIS, Prescribing Information System for community-based prescriptions; LES Area, local enhanced service area; ICD-10, International Classification of Disease, 10th Version; BNF, British National Formulary; PAD, peripheral arterial disease; CAD, coronary artery disease; HF, heart failure; ACEi, angiotensin-converting-enzyme inhibitor; ARB, angiotensin II receptor blocker; LD, loop diuretic; MRA, mineralocorticoid receptor antagonists.

Supplementary Table 2 Codes used to identify a history of CAD, PAD, HF, or a prescription for an ACEi, ARB, MRA, beta-blocker, or loop diuretics.

Inclusion reason	Code type	Code	Description
HF	See Supplementary Table 4		Heart Failure
ARB	See Supplementary Table 7		Angiotensin II receptor blockers
ACEi	See Supplementary Table 7		Angiotensin-converting-enzyme inhibitors
LD	See Supplementary Table 7		Loop diuretics
MRA	See Supplementary Table 7		Mineralocorticoid-receptor antagonists
Beta-blocker	See Supplementary Table 7		Beta-blockers
PAD	Read	14F7	H/O: arterial lower limb ulcer
	Read	14NB	H/O: Peripheral vascular disease procedure
	Read	2G63	Ischaemic toe
	Read	7A100	Emerg aortic bypass by anastomosis axillary to femoral art
	Read	7A101	Bypass aorta by anastomosis axillary to femoral artery NEC
	Read	7A102	Axillo-bifemoral bypass graft
	Read	7A103	Axillo-unifemoral PTFE bypass graft
	Read	7A12.00	Other bypass of bifurcation of aorta
	Read	7A120	Emerg bypass bifurc aorta by anast aorta to femoral artery
	Read	7A121	Bypass bifurc aorta by anastom aorta to femoral artery NEC
	Read	7A12111	Aorto bifemoral graft
	Read	7A12112	Dacron aortofemoral Y graft
	Read	7A123	Bypass bifurcation aorta by anastom aorta to iliac artery
	Read	7A12311	Aorto biiliac graft
	Read	7A12312	Dacron aortoiliac Y graft
	Read	7A12y	Other specified other bypass of bifurcation of aorta
	Read	7A12z	Other bypass of bifurcation of aorta NOS
	Read	7A192	Open embolectomy of bifurcation of aorta
	Read	7A41	Other bypass of iliac artery
	Read	7A410	Emerg bypass iliac art by iliac/femoral art anastomosis NEC
	Read	7A411	Bypass iliac artery by iliac/femoral artery anastomosis NEC
	Read	7A412	Emerg bypass iliac artery by femoral/femoral art anast NEC
	Read	7A41211	Emergency femoro-femoral prosthetic cross over graft
	Read	7A413	Bypass iliac artery by femoral/femoral art anastomosis NEC
	Read	7A41311	Femoro-femoral prosthetic cross over graft
	Read	7A414	Emerg bypass comm iliac art by aorta/com iliac art anast NEC
	Read	7A416	Emerg bypass leg artery by aorta/com fem art anastomosis NEC
	Read	7A419	Bypass common iliac artery by aorta/com iliac art anast NEC

	Read	7A41B	Bypass leg artery by aorta/com femoral art anastomosis NEC
	Read	7A41C	Bypass leg artery by aorta/deep femoral art anastomosis NEC
	Read	7A41F	Ilio-femoral prosthetic cross over graft
	Read	7A41y	Other specified other bypass of iliac artery
	Read	7A41z	Other bypass of iliac artery NOS
	Read	7A42	Reconstruction of iliac artery
	Read	7A420	Endarterectomy and patch repair of iliac artery
	Read	7A42011	Endarterectomy and patch repair of common iliac artery
	Read	7A42012	Iliac endarterectomy and patch
	Read	7A421	Endarterectomy of iliac artery NEC
	Read	7A42111	Endarterectomy of common iliac artery NEC
	Read	7A42y	Other specified reconstruction of iliac artery
	Read	7A42z	Reconstruction of iliac artery NOS
	Read	7A43	Other open operations on iliac artery
	Read	7A430	Repair of iliac artery NEC
	Read	7A43011	Repair of common iliac artery NEC
	Read	7A431	Open embolectomy of iliac artery
	Read	7A43111	Open embolectomy of common iliac artery
	Read	7A433	Open insertion of iliac artery stent
	Read	7A440	Percutaneous transluminal angioplasty of iliac artery
	Read	7A441	Percutaneous transluminal embolectomy of iliac artery
	Read	7A443	Insertion of iliac artery stent
	Read	7A444	Percutaneous transluminal insertion of iliac artery stent
	Read	7A44y	Other specified transluminal operation on iliac artery
	Read	7A44z	Transluminal operation on iliac artery NOS
	Read	7A47	Other emergency bypass of femoral artery or popliteal artery
	Read	7A470	Emerg bypass femoral art by fem/pop art anast c prosth NEC
PAD	Read	7A471	Emerg bypass popliteal art by pop/pop art anast c prosth NEC
	Read	7A472	Emerg bypass femoral art by fem/pop a anast c vein graft NEC
	Read	7A473	Emerg bypass pop art by pop/pop art anast c vein graft NEC
	Read	7A474	Emerg bypass femoral art by fem/tib art anast c prosth NEC
	Read	7A476	Emerg bypass femoral art by fem/tib a anast c vein graft NEC
	Read	7A477	Emerg bypass pop art by pop/tib art anast c vein graft NEC
	Read	7A47B	Emerg bypass pop art by pop/peron art anast c vein graft NEC
	Read	7A47C	Emerg bypass femoral artery by fem/fem art anastomosis NEC
	Read	7A47D	Emerg bypass popliteal artery by pop/fem art anastomosis NEC
	Read	7A47y	Other emergency bypass of femoral or popliteal artery OS
	Read	7A47z	Other emergency bypass of femoral or popliteal artery NOS
	Read	7A48	Other bypass of femoral artery or popliteal artery
	Read	7A480	Bypass femoral artery by fem/pop art anast c prosthesis NEC
	Read	7A481	Bypass popliteal artery by pop/pop a anast c prosthesis NEC
	Read	7A482	Bypass femoral artery by fem/pop art anast c vein graft NEC
	Read	7A483	Bypass popliteal artery by pop/pop a anast c vein graft NEC

	Read	7A484	Bypass femoral artery by fem/tib art anast c prosthesis NEC
	Read	7A485	Bypass popliteal artery by pop/tib a anast c prosthesis NEC
	Read	7A486	Bypass femoral artery by fem/tib art anast c vein graft NEC
	Read	7A487	Bypass popliteal artery by pop/tib a anast c vein graft NEC
	Read	7A488	Bypass femoral artery by fem/peron a anast c prosthesis NEC
	Read	7A48A	Bypass femoral artery by fem/peron a anast c vein graft NEC
	Read	7A48B	Bypass popliteal art by pop/peron art anast c vein graft NEC
	Read	7A48C	Bypass femoral artery by femoral/femoral art anastomosis NEC
	Read	7A48D	Bypass popliteal artery by pop/fem artery anastomosis NEC
	Read	7A48E	Femoro-femoral prosthetic cross over graft
	Read	7A48y	Other bypass of femoral artery or popliteal artery OS
	Read	7A48z	Other bypass of femoral artery or popliteal artery NOS
	Read	7A49	Reconstruction of femoral artery or popliteal artery
	Read	7A490	Endarterectomy and patch repair of femoral artery
	Read	7A491	Endarterectomy and patch repair of popliteal artery
	Read	7A492	Endarterectomy of femoral artery NEC
	Read	7A493	Endarterectomy of popliteal artery NEC
	Read	7A494	Profundoplasty femoral artery & patch repair deep fem artery
	Read	7A495	Profundoplasty and patch repair of popliteal artery
	Read	7A496	Profundoplasty of femoral artery NEC
	Read	7A497	Profundoplasty of popliteal artery NEC
	Read	7A498	Reconstruction of femoral artery with vein graft
	Read	7A499	Reconstruction of popliteal artery with vein graft
	Read	7A49y	Reconstruction of femoral or popliteal artery OS
	Read	7A49z	Reconstruction of femoral or popliteal artery NOS
	Read	7A4A	Other open operations on femoral artery or popliteal artery
	Read	7A4A0	Repair of femoral artery NEC
PAD	Read	7A4A1	Repair of popliteal artery NEC
	Read	7A4A2	Open embolectomy of femoral artery
	Read	7A4A211	Open thrombectomy of femoral artery
	Read	7A4A212	Open femoral embolectomy
	Read	7A4A3	Open embolectomy popliteal artery
	Read	7A4A311	Open thrombectomy of popliteal artery
	Read	7A4A7	Repair of femoral artery with temporary silastic shunt
	Read	7A4A8	Repair of popliteal artery with temporary silastic shunt
	Read	7A4Ay	Other open operation on femoral or popliteal artery OS
	Read	7A4B0	Percutaneous transluminal angioplasty of femoral artery
	Read	7A4B1	Percutaneous transluminal angioplasty of popliteal artery
	Read	7A4B2	Percutaneous transluminal embolectomy of femoral artery
	Read	7A4B3	Percutaneous transluminal embolectomy of popliteal artery
	Read	7A4B4	Percutaneous transluminal embolisation of femoral artery
	Read	7A4B5	Percutaneous transluminal embolisation of popliteal artery
	Read	7A4B8	Percut translum thrombolysis femoral graft streptokinase

PAD	Read	7A4B9	Percutaneous transluminal insertion of stent femoral artery
	Read	7A50	Revision of reconstruction of artery
	Read	7A500	Revision of reconstruction involving aorta
	Read	7A501	Revision of reconstruction involving iliac artery
	Read	7A502	Revision of reconstruction involving femoral artery
	Read	7A503	Revision of reconstruction of popliteal artery
	Read	A3A0F	Gas gangrene-foot
	Read	C107	Diabetes mellitus with peripheral circulatory disorder
	Read	C1070	Diabetes mellitus, juvenile +peripheral circulatory disorder
	Read	C1071	Diabetes mellitus, adult, + peripheral circulatory disorder
	Read	C1073	IDDM with peripheral circulatory disorder
	Read	C1074	NIDDM with peripheral circulatory disorder
	Read	C107z	Diabetes mellitus NOS with peripheral circulatory disorder
	Read	C108G	Insulin dependent diab mell with peripheral angiopathy
	Read	C109F	Non-insulin-dependent d m with peripheral angiopath
	Read	C109F11	Type II diabetes mellitus with peripheral angiopathy
	Read	C109F12	Type 2 diabetes mellitus with peripheral angiopathy
	Read	C10EG	Type 1 diabetes mellitus with peripheral angiopathy
	Read	C10FF	Type 2 diabetes mellitus with peripheral angiopathy
	Read	G700	Aorto-iliac disease
	Read	G702	Extremity artery atheroma
	Read	G702z	Extremity artery atheroma NOS
	Read	G73	Other peripheral vascular disease
	Read	G731	Thromboangiitis obliterans
	Read	G7310	Buerger's disease
	Read	G731z	Thromboangiitis obliterans NOS
	Read	G732	Peripheral gangrene
	Read	G7320	Gangrene of toe
	Read	G7321	Gangrene of foot
	Read	G733	Ischaemic foot
	Read	G73y	Other specified peripheral vascular disease
	Read	G73y0	Diabetic peripheral angiopathy
	Read	G73y1	Peripheral angiopathic disease EC NOS
	Read	G73y2	Acrocyanosis
	Read	G73y4	Acroparaesthesia - Schultze's type
	Read	G73y5	Acroparaesthesia - Nothnagel's type
	Read	G73y511	Nothnagel's vasomotor acroparaesthesia
	Read	G73y6	Acroparaesthesia - unspecified
	Read	G73y7	Erythrocyanosis
	Read	G73y8	Erythromelalgia
	Read	G73yz	Other specified peripheral vascular disease NOS
	Read	G73z	Peripheral vascular disease NOS
	Read	G73z0	Intermittent claudication

PAD	Read	G73z011	Claudication
	Read	G73zz	Peripheral vascular disease NOS
	Read	G740	Aortoiliac obstruction
	Read	G7424	Embolism and thrombosis of the femoral artery
	Read	G7425	Embolism and thrombosis of the popliteal artery
	Read	G7426	Embolism and thrombosis of the anterior tibial artery
	Read	G7427	Embolism and thrombosis of the dorsalis pedis artery
	Read	G7429	Embolism and thrombosis of a leg artery NOS
	Read	G742z	Peripheral arterial embolism and thrombosis NOS
	Read	G74y0	Embolism and/or thrombosis of the common iliac artery
	Read	G74y1	Embolism and/or thrombosis of the internal iliac artery
	Read	G74y2	Embolism and/or thrombosis of the external iliac artery
	Read	G74y3	Embolism and thrombosis of the iliac artery unspecified
	Read	Gyu74	[X]Other specified peripheral vascular diseases
	Read	M271	Ischaemic leg ulcer
	Read	M2710	Ischaemic ulcer diabetic foot
	Read	M2713	Arterial leg ulcer
	Read	M2714	Mixed venous and arterial leg ulcer
	Read	R0542	[D]Gangrene of toe in diabetic
	Read	R0543	[D]Widespread diabetic foot gangrene
	Read	R0550	[D]Failure of peripheral circulation
	Read	R055011	[D]Peripheral circulatory failure
	ICD-10	I731	Thromboangiitis obliterans [Buerger]
	ICD-10	I738	Other specified peripheral vascular diseases
	ICD-10	I739	Peripheral vascular disease, unspecified
	ICD-10	I743	Embolism and thrombosis of arteries of lower extremities
	ICD-10	I744	Embolism and thrombosis of arteries of extremities, unspecified
	ICD-10	I745	Embolism and thrombosis of iliac artery
	OPCS-4	L50	Other emergency bypass of iliac artery
	OPCS-4	L51	Other bypass of iliac artery
	OPCS-4	L52	Reconstruction of iliac artery
	OPCS-4	L530	Other open operations on iliac artery
	OPCS-4	L531	Repair of iliac artery NEC
	OPCS-4	L532	Open embolectomy of iliac artery
	OPCS-4	L541	Percutaneous transluminal angioplasty of iliac artery
	OPCS-4	L542	Percutaneous transluminal embolectomy of iliac artery
	OPCS-4	L544	Percutaneous transluminal insertion of stent into iliac artery
	OPCS-4	L548	Other specified transluminal operations on iliac artery
	OPCS-4	L549	Unspecified transluminal operations on iliac artery
	OPCS-4	L58	Other emergency bypass of femoral artery
	OPCS-4	L59	Other bypass of femoral artery
	OPCS-4	L60	Reconstruction of femoral artery
	OPCS-4	L620	Other open operations on femoral artery

	OPCS-4	L621	Repair of femoral artery NEC
	OPCS-4	L622	Open embolectomy of femoral artery
	OPCS-4	L628	Other specified other open operations on femoral artery
	OPCS-4	L629	Unspecified other open operations on femoral artery
	OPCS-4	L631	Percutaneous transluminal angioplasty of femoral artery
	OPCS-4	L632	Percutaneous transluminal embolectomy of femoral artery
	OPCS-4	L633	Percutaneous transluminal embolisation of femoral artery
	OPCS-4	L635	Percutaneous transluminal insertion of stent into femoral artery
	OPCS-4	L650	Revision of reconstruction of artery
	OPCS-4	L651	Revision of reconstruction involving aorta
	OPCS-4	L652	Revision of reconstruction involving iliac artery
	OPCS-4	L653	Revision of reconstruction involving femoral artery
CAD	ICD-10	I20	Angina pectoris
	ICD-10	I21	Acute myocardial infarction
	ICD-10	I22	Subsequent myocardial infarction
	ICD-10	I23	Certain current complications following acute myocardial infarction
	ICD-10	I24	Other acute ischaemic heart diseases
	ICD-10	I25	Chronic ischaemic heart disease
	Read	14AL	H/O: Treatment for ischaemic heart disease
	Read	G310	Dressler's syndrome
	Read	G33z5	Post infarct angina
	Read	889A	Diab mellit insulin-glucose infus acute myocardial infarct
	Read	6A2	Coronary heart disease annual review
	Read	6A4	Coronary heart disease review
	Read	8B3k	Coronary heart disease medication review
	Read	8H2V	Admit ischaemic heart disease emergency
	Read	G3	Ischaemic heart disease
	Read	G31	Other acute and subacute ischaemic heart disease
	Read	G3110	Myocardial infarction aborted
	Read	G311011	MI - myocardial infarction aborted
	Read	G31y	Other acute and subacute ischaemic heart disease
	Read	G31y2	Subendocardial ischaemia
	Read	G31y3	Transient myocardial ischaemia
	Read	G31yz	Other acute and subacute ischaemic heart disease NOS
	Read	G34	Other chronic ischaemic heart disease
	Read	G340	Coronary atherosclerosis
	Read	G344	Silent myocardial ischaemia
	Read	G34y	Other specified chronic ischaemic heart disease
	Read	G34y1	Chronic myocardial ischaemia
	Read	G34yz	Other specified chronic ischaemic heart disease NOS
	Read	G34z	Other chronic ischaemic heart disease NOS
	Read	G34z0	Asymptomatic coronary heart disease
	Read	G3y	Other specified ischaemic heart disease

CAD	Read	G3z	Ischaemic heart disease NOS
	Read	Gyu3	[X]Ischaemic heart diseases
	Read	Gyu32	[X]Other forms of acute ischaemic heart disease
	Read	Gyu33	[X]Other forms of chronic ischaemic heart disease
	Read	G300	Acute anterolateral infarction
	Read	G301	Other specified anterior MI
	Read	G3010	Acute anteroapical infarction
	Read	G3011	Acute anteroseptal infarction
	Read	G301z	Anterior MI NOS
	Read	G302	Acute inferolateral infarction
	Read	G303	Acute inferoposterior infarction
	Read	G304	Posterior MI NOS
	Read	G305	Lateral MI NOS
	Read	G306	True posterior MI
	Read	G307	Acute subendocardial infarction
	Read	G3070	Acute non-Q wave infarction
	Read	G3071	Acute non-ST segment elevation MI
	Read	G308	Inferior MI NOS
	Read	G309	Acute Q-wave infarct
	Read	G30A	Mural thrombosis
	Read	G30B	Acute posterolateral MI
	Read	G30X	Acute transmural MI of unspecif site
	Read	G30X0	Acute ST segment elevation MI
	Read	G30y	Other acute MI
	Read	G30z	Acute MI NOS
	Read	G311	Unstable angina
	Read	G3112	Angina at rest
	Read	G3113	Refractory angina
	Read	G3114	Worsening angina
	Read	G3115	Acute coronary syndrome
	Read	G31y0	Acute coronary syndrome
	Read	G33	Angina pectoris
	Read	G33z	Angina pectoris NOS
	Read	G33z3	Angina on effort
	Read	G33z4	Ischaemic chest pain
	Read	G33zz	Angina pectoris NOS
	Read	G3400	Single coronary vessel disease
	Read	G3401	Double coronary vessel disease
	Read	G70y011	Carteroid artery disease
	Read	G30	Acute MI
	Read	G32	Old MI
	Read	G32	Old MI

ICD-10, International Classification of Disease, 10th Version; BNF, British National Formulary; PAD, peripheral arterial disease; CAD, coronary artery disease; HF, heart failure; ACEi, angiotensin-converting-enzyme inhibitor; ARB, angiotensin II receptor blocker; LD, loop diuretic; MRA, mineralocorticoid receptor antagonists.

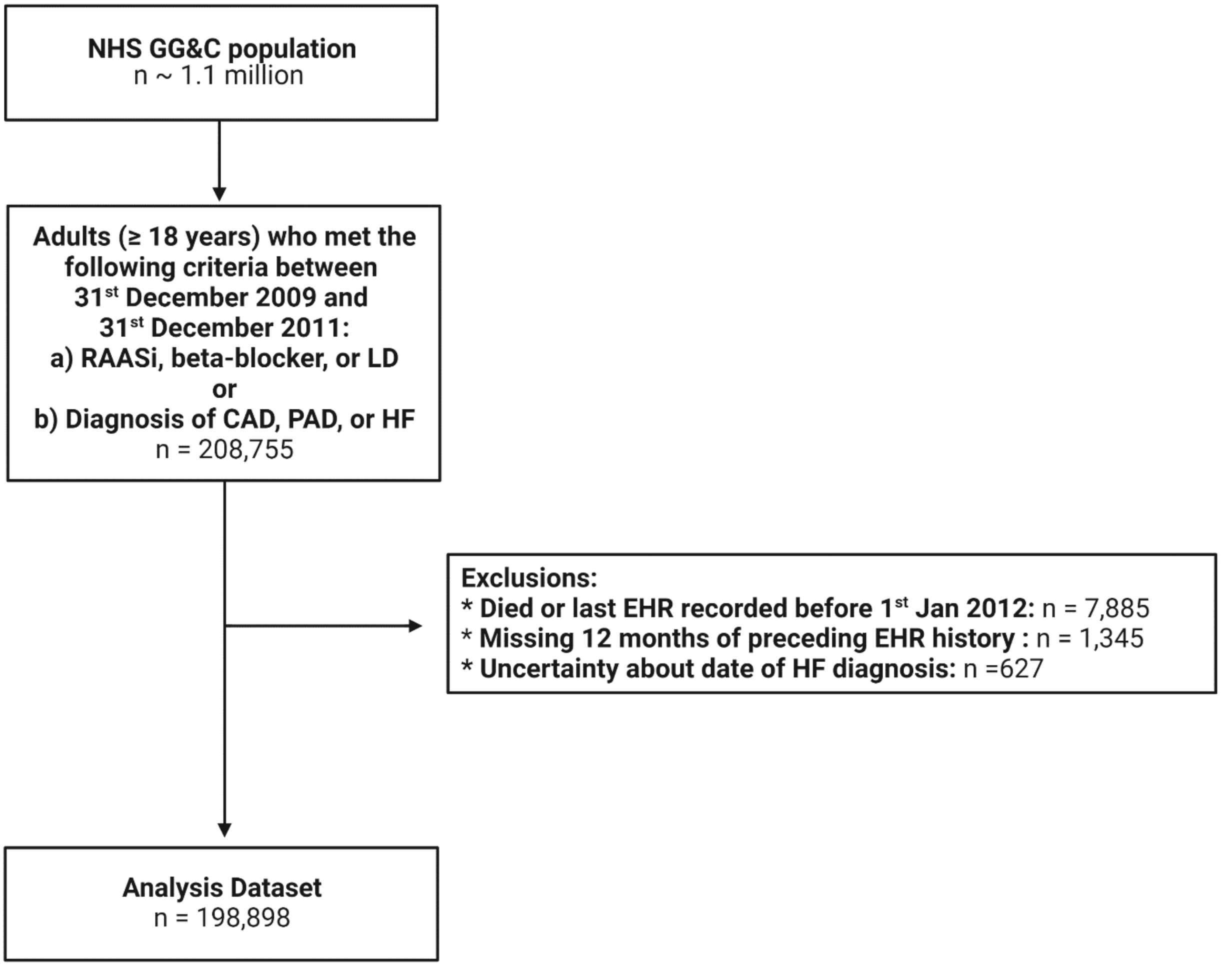

Supplementary Figure 1 Flow chart of inclusion and exclusion of NHS Greater Glasgow & Clyde patients

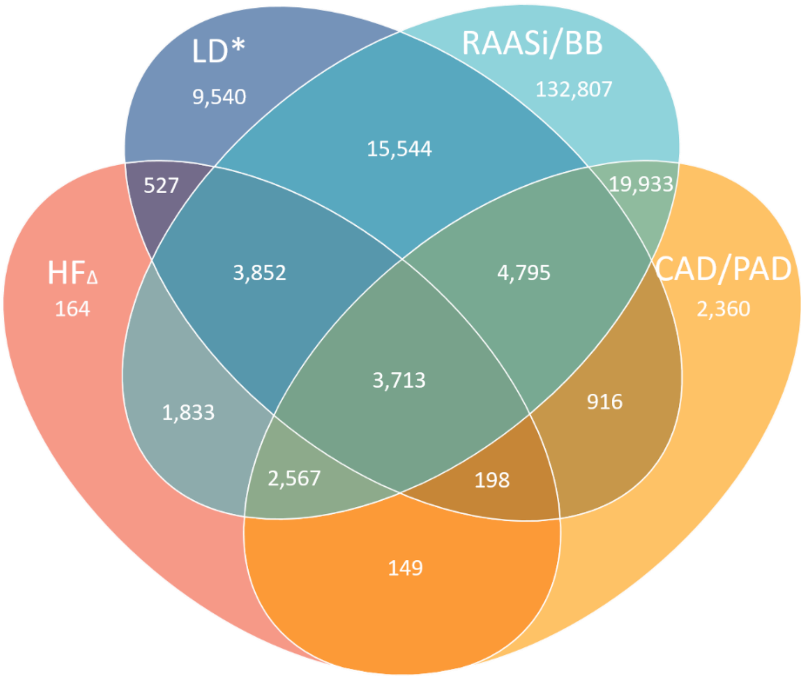

Supplementary Figure 1 Venn diagram of patients' status on cohort inclusion on 31 December 2011

HF, heart failure; LD, loop diuretics, BB, beta-blocker; RAASi, renin–angiotensin–aldosterone system inhibitors; CAD, coronary artery disease; PAD, peripheral arterial disease.

* LD includes those with either a single or a repeat prescription of a loop diuretic.

Δ HF includes diagnosis records from 2000 and <6 individuals who subsequently did not survive their index admission.

Data sources and coverage

Supplementary Table 3 Notes on data sources used for analysis, including date ranges and coverage.

Data source	Description	Notes	Date Ranges	Coverage
SMR00	Outpatient attendance	Diagnostic codes for neurological and mental conditions only	2000 onwards	≥99%(4)
SMR01	General/acute inpatient & day cases	Used to define comorbidities. Data are stored as episodes of care, which can contain up to 6 discharge codes and up to 4 procedures. Patients receive a new episode of care each time they change specialty, significant facility, or consultant for medical reasons (5). Continuous admissions were defined as the set of all episodes of care that differed by no more than one calendar day between one episode's discharge and the next episode's admission, allowing for hospital transfers around midnight to be counted as one continuous admission.	2000 onwards	≥99%(4)
SMR04	Mental health inpatient & day cases		2000 onwards	≥99%

The Scottish National Prescribing Information System	Community-based prescribing	Contains all general practice and hospital-based prescriptions dispensed within the community.	31 December 2009 onwards	≥99%
SCI Store	Scottish Care Information (SCI) Store for laboratory data	Haematology and biochemistry laboratory data from primary and secondary care	2010 onwards	≥99%
Xcelera & EchoPAC	Echocardiography reports	Greater Glasgow & Clyde Health Board reports produced on the two systems	Records start in 2010, but full coverage begins on 1 January 2012	Greater Glasgow & Clyde Health Board secondary care
MUSE	ECG reports	Greater Glasgow & Clyde Health Board generated automatically generated ECG measurements, including QRS duration and rhythm from MUSE-compatible electrocardiographs	Records start in 2010, but full coverage begins on 1 January 2012	Greater Glasgow & Clyde Health Board secondary care
Deaths	Death Records	Death records, including date of death and cause of death	2010 onwards	≥99%
GP LES	General Practice Local Enhanced Services	Enhanced service areas include coronary heart disease, diabetes, heart failure, stroke, chronic obstructive pulmonary disease (COPD), left ventricular systolic dysfunction (LVSD), learning disability, KeepWell, alcohol misuse, individuals in care homes, and nationally enhanced services for drug misuse. Each record contains, at most, one diagnostic code.	2000 onwards	Within the Greater Glasgow & Clyde Health Board, 82.8% of surgeries provided data to General Practice Local Enhanced Services

Classification of heart failure, medications, socioeconomic deprivation, and co-morbidities

Patients were considered to have a diagnosis if a code was recorded in any diagnostic or procedural position, where applicable.

Supplementary Table 4 ICD-10 and NHS Greater Glasgow & Clyde formatted Read Codes used to identify patients with heart failure.

Code Type	Code	Description	Conrad et al.(6)	Included
ICD-10	I50	Heart Failure	Yes	Yes
ICD-10	I420	Dilated cardiomyopathy (congestive cardiomyopathy)	Yes	Yes
ICD-10	I429	Cardiomyopathy, unspecified (Cardiomyopathy (primary)(secondary) NOS)	Yes	Yes
ICD-10	I110	Hypertensive heart disease with (congestive) heart failure	Yes	Yes
ICD-10	I255	Ischaemic cardiomyopathy	Yes	Yes
ICD-10	I132	Hypertensive heart and renal disease with both (congestive) heart failure and renal failure	Yes	Yes
ICD-10	I130	Hypertensive heart and renal disease with (congestive) heart failure	Yes	Yes
Read	585f	Echocardiogram shows left ventricular systolic dysfunction	Yes	Yes
Read	G58	Heart Failure	Yes	Yes
Read	G581	Left ventricular failure	Yes	Yes
Read	662g	NYHA classification class II	Yes	Yes
Read	662f	NYHA classification - class I	Yes	Yes
Read	G5yy9	Left ventricular systolic dysfunction	Yes	Yes
Read	G580	Congestive heart failure	Yes	Yes
Read	662h	NYHA classification- class III	Yes	Yes
Read	9hH1	Excepted heart failure quality indicators: informed dissent	Yes	Yes
Read	9hH0	Excepted heart failure quality indicators: patient unsuitable	Yes	Yes
Read	21264	Heart Failure resolved	Yes	Yes
Read	G5802	Decompensated cardiac failure	Yes	Yes
Read	G58z	Heart failure NOS	Yes	Yes
Read	662i	NYHA classification - class IV	Yes	Yes
Read	G5801	Chronic congestive heart failure	Yes	Yes
Read	8HHb	Referral to heart failure nurse	Yes	Yes
Read	G5810	Acute left ventricular failure	Yes	Yes
Read	G343	Ischaemic cardiomyopathy	Yes	Yes
Read	G582	Acute heart failure	Yes	Yes
Read	G5800	Acute congestive heart failure	No	Yes
Read	G583	Heart failure with normal ejection fraction	Yes	Yes
Read	585g	Echocardiogram shows left ventricular diastolic dysfunction	Yes	Yes
Read	G584	Right ventricular failure	Yes	Yes
Read	G5yyA	Left ventricular diastolic dysfunction	Yes	Yes
Read	G5544	Primary dilated cardiomyopathy	Yes	Yes
Read	G55z	Cardiomyopathy NOS	Yes	Yes
Read	G5803	Compensated cardiac failure	Yes	Yes
Read	G5804	Congestive heart failure due to valvular disease	Yes	Yes
Read	G555	Alcoholic cardiomyopathy	Yes	Yes
Read	G551	Hypertrophic obstructive cardiomyopathy	Yes	Yes

Read	G5yyD	Left ventricular cardiac dysfunction	Yes	Yes
Read	9hH	Exception reporting heart failure quality indicators	Yes	Yes
Read	G1yz1	Pneumatic left ventricular failure	Yes	Yes
Read	679X	Heart failure education	Yes	Yes
Read	8HBE	Heart failure follow-up	Yes	Yes
Read	1O1	Heart failure confirmed	Yes	Yes
Read	8H2S	Admit heart failure emergency	Yes	Yes
Read	8CMK	Has heart failure management plan	Yes	Yes
Read	9Or5	Heart failure monitoring third letter	Yes	Yes
Read	9Or3	Heart failure monitoring first letter	Yes	Yes
Read	9Or4	Heart failure monitoring second letter	Yes	Yes
Read	9Or2	Heart failure monitoring verbal invite	Yes	Yes
Read	9Or	Heart failure monitoring administration	Yes	Yes
Read	9Or1	Heart failure monitoring telephone invite	Yes	Yes
Read	8HgD	Discharge from heart failure nurse service	Yes	Yes
Read	8HHz	Referral to heart failure exercise programme	Yes	Yes
Read	8CL3	Heart failure care plan discussed with patient	Yes	Yes
Read	8IE1	Referral to heart failure exercise program declined	Yes	Yes
Read	8IB8	Referral to heart failure exercise program not indicated	Yes	Yes
Read	8CeC	Preferred place of care for next exacerbation heart failure	Yes	Yes
Read	G55	Cardiomyopathy	Yes	Yes
Read	G5540	Congestive Cardiomyopathy	Yes	Yes
Read	G559	Arrhythmogenic right ventricular cardiomyopathy	Yes	Yes
Read	33BA	Impaired left ventricular function	Yes	Yes
Read	G41z	Chronic cor pulmonale	Yes	Yes
Read	G5543	Hypertrophic non-obstructive cardiomyopathy	Yes	Yes
Read	G55y	Secondary cardiomyopathy NOS	Yes	Yes
Read	G5542	Familial cardiomyopathy	Yes	Yes
Read	8CMW8	Heart failure clinical pathway	Yes	Yes
Read	G5581	Cardiomyopathy in myotonic dystrophy	Yes	Yes
Read	Gyu5M	[X] Other hypertrophic cardiomyopathy	Yes	Yes
Read	G2111	Benign hypertensive heart disease with CCF	Yes	Yes
Read	G21z1	Hypertensive heart disease NOS with CCF	Yes	Yes
Read	G232	hypertensive heart and renal disease with (congestive) heart failure	Yes	Yes
Read	662T	Congestive heart failure monitoring	Yes	Yes
Read	662W	Heart failure annual review	Yes	Yes
Read	662p	Heart failure 6 month review	Yes	Yes
Read	9On	Left ventricular dysfunction monitoring administration	Yes	Yes
Read	9On0	Left ventricular dysfunction monitoring 1st letter	Yes	Yes
Read	9On1	Left ventricular dysfunction monitoring 2nd letter	Yes	Yes
Read	9On2	Left ventricular dysfunction monitoring 3rd letter	Yes	Yes
Read	9On3	Left ventricular dysfunction monitoring verbal invite	Yes	Yes
Read	9On4	Left ventricular dysfunction monitoring telephone invite	Yes	Yes
Read	9N4s	Did not attend practice nurse heart failure clinic	Yes	Yes
Read	8B29	Cardiac failure therapy	Yes	Yes
Read	23E1	O/E -pulmonary oedema	Yes	Yes
Read	9h1	Exception reporting: LVD quality indicators	Yes	Yes
Read	9h12	Excepted from LVD quality indicators: informed dissent	Yes	Yes

Read	9h11	Excepted from LVD quality indicators: Patient unsuitable	Yes	Yes
Read	14A6	H/O: heart failure	Yes	Yes
Read	14AM	H/O: heart failure in last year	Yes	Yes
Read	8HTL0	Referral to rapid access heart failure clinic	Yes	Yes
Read	8IE0	Referral to heart failure education group declined	Yes	Yes
Read	G234	Hyperten heart & renal dis + both (congestv) heart and renal fail	No	Yes
Read	12CR	HF: Hypertrophic obstructive cardiomyopathy	Yes	Yes

Following previously published methodologies, cases where the first recorded diagnostic code referred to an existing diagnosis were excluded from incident analysis, as the date of diagnosis is unknown (6).

Supplementary Table 5 NHS Greater Glasgow & Clyde formatted Read Codes used to exclude heart failure cases where the code refers to an existing diagnosis after 31 December 2011.

Code	Description	Conrad et al.	Included
9N4s	Did not attend practice nurse heart failure clinic	Yes	Yes
8B29	Cardiac failure therapy	Yes	Yes
679X	Heart failure education	Yes	Yes
8HHb	Referral to heart failure nurse	Yes	Yes
23E1	O/E – pulmonary oedema	Yes	Yes
9hH	Exception reporting: heart failure quality indicators	Yes	Yes
9hH0	Excepted heart failure quality indicators: Patient unsuitabl	Yes	Yes
9h1	Exception reporting: LVD quality indicators	Yes	Yes
9h11	Exception from LVD quality indicators: Patient unsuitable	Yes	Yes
9h12	Exception from LVD quality indicators: Informed dissent	Yes	Yes
14A6	H/O: heart failure	Yes	Yes
14AM	H/O: Heart failure in last year	Yes	Yes
662p	Heart failure 6 month review	Yes	Yes
662T	Congestive heart failure monitoring	Yes	Yes
662W	Heart failure annual review	Yes	Yes
8CL3	Heart failure care plan discussed with patient	Yes	Yes
8HBE	Heart failure follow-up	Yes	Yes
8HHz	Referral to heart failure exercise programme	Yes	Yes
8Hk0	Referred to heart failure education group	Yes	Yes
9hH1	Excepted heart failure quality indicators: Informed dissent	Yes	Yes
9Or	Heart failure monitoring administration	Yes	Yes
9Or0	Heart failure review completed	Yes	Yes
9Or1	Heart failure monitoring telephone invite	Yes	Yes
9Or2	Heart failure monitoring verbal invite	Yes	Yes
9Or3	Heart failure monitoring first letter	Yes	Yes
9Or4	Heart failure monitoring second letter	Yes	Yes
9Or5	Heart failure monitoring third letter	Yes	Yes
9On	Left ventricular dysfunction monitoring administration	Yes	Yes
9On0	Left ventricular dysfunction monitoring first letter	Yes	Yes
9On1	Left ventricular dysfunction monitoring second letter	Yes	Yes
9On2	Left ventricular dysfunction monitoring third letter	Yes	Yes
9On3	Left ventricular dysfunction monitoring verbal invite	Yes	Yes
9On4	Left ventricular dysfunction monitoring telephone invite	Yes	Yes
2126400	Heart Failure Resolved	Yes	Yes

21264	Heart Failure Resolved	Yes	Yes
8HgD	Discharged from heart failure nurse service	Yes	Yes
8HTL000	Referral to rapid access heart failure clinic	Yes	Yes
8HTL0	Referral to rapid access heart failure clinic	Yes	Yes
8IB8	Referral to heart failure exercise programme not indicated	Yes	Yes
8IE1	Referral to heart failure excursive programme declined	Yes	Yes
81E0	Referral to heart failure education group declined	Yes	Yes
12CR	FH: Hypertrophic obstructive cardiomyopathy	Yes	Yes

Supplementary Table 6 Description of how co-morbidities were defined and the data sources used to define them.

Co-morbidity	Defining the history of co-morbidity	Data sources used
Hypertension	CALIBER phenotype	SMR01, SMR04, & GP LES
Diabetes mellitus	A record of diabetes as defined by the CALIBER phenotype	SMR01, SMR04, & GP LES
Thyroid disease	A record of hyperthyroidism or hypothyroidism as defined by CALIBER phenotype	SMR01, SMR04, & GP LES
Atrial fibrillation or flutter	Modified from the CALIBER phenotype to only include cases where there was a coded record of a diagnosis	SMR01, SMR04, & GP LES
Ischaemic heart disease	A combination of codes identified in previously published research (7) and CALIBER definitions of myocardial infarction, coronary thrombosis, unstable angina, stable angina, and coronary artery disease.	SMR01, SMR04, & GP LES
Myocardial infarction	Record of at least one of the following based on CALIBER phenotype: acute myocardial infarction, complications of myocardial infarction, subsequent myocardial infarction, or not otherwise specified myocardial infarction.	SMR01, SMR04, & GP LES
Valve disease	CALIBER phenotype	SMR01, SMR04, & GP LES
Peripheral arterial disease	CALIBER phenotype	SMR01, SMR04, & GP LES
Stroke	Record of at least one of the following based on CALIBER phenotype: intracerebral haemorrhage, ischaemic stroke, stroke not elsewhere specified, or subarachnoid haemorrhage.	SMR01, SMR04, & GP LES
Chronic obstructive pulmonary disease	CALIBER phenotype	SMR01, SMR04, & GP LES
Cancer	CALIBER phenotype	SMR01, SMR04, & GP LES
Liver disease	CALIBER phenotype was used for the combination of liver fibrosis, sclerosis, and cirrhosis. Codes were only found in SMR01 and SMR04.	SMR01, SMR04, & GP LES
Dementia	CALIBER phenotype includes dementia in Alzheimer's disease, vascular or arteriosclerotic dementia, dementia in other diseases or causes, and unspecified dementia.	SMR0, SMR01, SMR04, & GP LES

All medications prescribed and dispensed within Scotland for individuals within the cohort have been extracted from the Scottish Prescribing Information System (8). Medications prescribed within the NHS are contained within the British National Formulary (BNF) and are assigned a hierarchical code to identify the product's chapter, section, and paragraph. See https://openprescribing.net/bnf/ for a complete list of medications. Patients were considered to be on a medication if they had a prescription dispensed within the 180 days leading up to 1 January 2012.

Supplementary Table 7 Prescribed medication classifications, categories each medication was assigned to, the portion of the BNF code used to select for the category, and the description of the BNF selection code.

Category	BNF selection code	Description

Alpha-adrenoreceptor blocking agents	020504	Alpha-adrenoceptor blocking agents
ACEi	0205051	Angiotensin-converting enzyme
ARB	0205052<br>0206020Z0	Angiotensin receptor blockers<br>Valsartan/Amlodipine
Beta-blocker	0204	Beta-adrenergicblockingagents
Bronchodilators	0301	Bronchodilators
Calcium-channel blockers	020602<br>0205052AB<br>0205052AC	Calcium-channel blockers<br>Olmesartan medoxomil/amlodipine<br>Olmesartan medoxomil/amlodipine/hydrochlorothiazide
	0206020C0<br>0206020T0	Diltiazem hydrochloride<br>Verapamil hydrochloride
Centrally-acting antihypertensives	020502	Centrally-acting antihypertensive agents
Digoxin	0201010F	Digoxin
Hypoglycemics	060101	Insulin
	060102	Other hypoglycaemics
Lipid-regulating	0212	Lipid-regulating
Loop diuretics	020202<br>0202040D0<br>0202040B0<br>0202040T0<br>0202040U0<br>0202080D0<br>0202080C0<br>0202080K0	Loop diuretics<br>Amiloride HCl with loop diuretics<br>Co-amilofruse (Amiloride hydrochloride/frusemide)<br>Spironolactone with loop diuretics<br>Triamterene with loop diuretics<br>Bumetanide/Amiloride hydrochloride<br>Bumetanide/potassium<br>Furosemide/potassium
Low dose aspirin	0209000A0<br>0204000AC<br>0209000V0	Aspirin<br>Bisoprolol fumarate/aspirin<br>Dipyridamole and Aspirin
MRA	0202030X0<br>0202030S0<br>0202040G0<br>0202040T0<br>0202040S0	Eplerenone<br>Spironolactone<br>Co-flumactone (Hydroflumethiazide/spironolactone)<br>Spironolactone with loop diuretics<br>Spironolactone with thiazides
NSAIDs	100101	Non-steroidal anti-inflammatory drugs
Oral anticoagulants	020802	Oral anticoagulants
Thiazides and related	020201<br>0202040H<br>0202040C0<br>0204000Y0<br>020400040<br>0205051AB<br>0202040A0<br>0202040S0<br>0202040V0<br>0202080B0<br>0205052Y0<br>020400010<br>020400030<br>0204000F0<br>0204000Q0<br>0204000W0<br>0205051H0<br>0205051K0<br>0205051N0<br>0205051P0<br>0205051Z0	Thiazides and related<br>Co-triamterzide (Triamterene/hydrochlorothiazide)<br>Co-amilozide (Amiloride hydrochloride/hydrochlorothiazide)<br>Co-prenozide (Oxprenolol hydrochloride/ cyclopenthiazide)<br>Co-tenidone (Atenolol/chlortalidone)<br>Perindopril tosilate/indapamide<br>Amiloride hydrochloride with thiazides<br>Spironolactone with thiazides<br>Triamterene with thiazides<br>Bendroflumethiazide/potassium<br>Olmesartan medoxomil/hydrochlorothiazide<br>Pindolol with diuretic<br>Timolol with diuretic<br>Atenolol with diuretic<br>Propranolol hydrochloride with diuretic<br>Metoprolol tartrate with diuretic<br>Enalapril maleate with diuretic<br>Lisinopril with diuretic<br>Perindopril erbumine with diuretic<br>Quinapril hydrochloride with diuretic<br>Perindopril arginine with diuretic

	0205052A0	Irbesartan with diuretic
	0205052P0	Losartan potassium with diuretic
	0205052R0	Telmisartan with diuretic
	0205052X0	Valsartan with diuretic
	0205052AC	Olmesartan medoxomil/amlodipine/ hydrochlorothiazide
	0205051G0	Co-zidocapt (Hydrochlorothiazide/captopril)
Thyroid disorders	060201	Thyroid hormones
	0602020D<br>0602020G<br>0602020N	Antithyroid drugs
BNF, British National Formulary; ACEi, Angiotensin-converting enzyme inhibitors; ARB, Angiotensin II receptor blocker; MRA, Mineralocorticoid receptor antagonists; NSAIDs, Non-steroidal anti-inflammatory drugs.		

Scottish Index of Multiple Deprivation

The Scottish Index of Multiple Deprivation is an area-based measurement of socioeconomic deprivation assigned to residents of Scotland based on where they live. Scottish residents' 2012 status was calculated by the Scottish Government using thirty-one indicators from seven different aspects of deprivation: income, employment, health, education, housing, geographic access, and crime. The indicators are combined using a weighted sum to create a single index, providing a relative ranking for each small geographic area in Scotland. Areas average about 800 individuals (9). It is important to note that the index can only measure an area's level of deprivation, not an individual's level of deprivation; the absence of deprivation should not necessarily be correlated with affluence. The terms most deprived or least deprived were used to refer to the areas and not to the individuals living in those areas (9).

R packages

The following R packages were the backbone of the analysis: tidyverse (10), survival (11), survminer (12), RODBC (13), viridis (14), cmprsk (15), lubridate (16), broom (17), ggfortify (18), forcats (19), gridExra (20), ggpubr (21), and reshape2 (22).

Aggregated data

NHS Greater Glasgow & Clyde Health Board population estimates based on revised National Records of Scotland mid-year population estimates by pre-April 2014 NHS Board areas (23).

Supplementary Table 8 Cohort group by age and sex against the estimated mid-year NHS Greater Glasgow & Clyde Health (GG&C) Board's mid-year 2012 population estimate.

Cohort Group	Sex	Age group	Number	GG&C population estimate	Percent of GG&C population
Neither	Women	18-29	4,799	110,408	4.35
Neither	Women	30-34	2,722	42,252	6.44
Neither	Women	35-39	3,211	37,934	8.46
Neither	Women	40-44	4,734	45,989	10.29
Neither	Women	45-49	6,694	48,958	13.67
Neither	Women	50-54	8,134	45,928	17.71
Neither	Women	55-59	8,544	38,388	22.26
Neither	Women	60-64	9,306	34,072	27.31
Neither	Women	65-69	9,326	30,156	30.93
Neither	Women	70-74	9,524	25,812	36.90
Neither	Women	75-79	8,742	23,234	37.63
Neither	Women	80-84	6,361	17,699	35.94
Neither	Women	85-89	3,440	10,473	32.85
Neither	Women	90+	1,485	6,103	24.33
Neither	Men	18-29	2,190	108,388	2.02

Neither	Men	30-34	1,450	40,760	3.56
Neither	Men	35-39	1,987	36,359	5.46
Neither	Men	40-44	3,637	42,110	8.64
Neither	Men	45-49	5,885	44,819	13.13
Neither	Men	50-54	8,589	42,508	20.21
Neither	Men	55-59	9,660	36,476	26.48
Neither	Men	60-64	10,572	32,068	32.97
Neither	Men	65-69	9,670	27,327	35.39
Neither	Men	70-74	8,378	20,536	40.80
Neither	Men	75-79	6,616	16,162	40.94
Neither	Men	80-84	4,117	10,555	39.01
Neither	Men	85-89	1,653	4,935	33.50
Neither	Men	90+	509	1,976	25.76
LD Only	Women	18-29	37	110,408	0.03
LD Only	Women	30-34	62	42,252	0.15
LD Only	Women	35-39	121	37,934	0.32
LD Only	Women	40-44	286	45,989	0.62
LD Only	Women	45-49	513	48,958	1.05
LD Only	Women	50-54	756	45,928	1.65
LD Only	Women	55-59	997	38,388	2.60
LD Only	Women	60-64	1,345	34,072	3.95
LD Only	Women	65-69	1,630	30,156	5.41
LD Only	Women	70-74	2,158	25,812	8.36
LD Only	Women	75-79	2,792	23,234	12.02
LD Only	Women	80-84	2,714	17,699	15.33
LD Only	Women	85-89	1,938	10,473	18.50
LD Only	Women	90+	1,426	6,103	23.37
LD Only	Men	18-29	10	108,388	0.01
LD Only	Men	30-34	12	40,760	0.03
LD Only	Men	35-39	49	36,359	0.13
LD Only	Men	40-44	111	42,110	0.26
LD Only	Men	45-49	178	44,819	0.40
LD Only	Men	50-54	308	42,508	0.72
LD Only	Men	55-59	463	36,476	1.27
LD Only	Men	60-64	750	32,068	2.34
LD Only	Men	65-69	929	27,327	3.40
LD Only	Men	70-74	1,120	20,536	5.45
LD Only	Men	75-79	1,219	16,162	7.54
LD Only	Men	80-84	1,032	10,555	9.78
LD Only	Men	85-89	688	4,935	13.94
LD Only	Men	90+	319	1,976	16.14
HF Only	Women	18-29	19	110,408	0.02
HF Only	Women	30-34	11	42,252	0.03
HF Only	Women	35-39	19	37,934	0.05
HF Only	Women	40-44	24	45,989	0.05
HF Only	Women	45-49	59	48,958	0.12
HF Only	Women	50-54	94	45,928	0.20
HF Only	Women	55-59	121	38,388	0.32
HF Only	Women	60-64	144	34,072	0.42

HF Only	Women	65-69	203	30,156	0.67
HF Only	Women	70-74	232	25,812	0.90
HF Only	Women	75-79	277	23,234	1.19
HF Only	Women	80-84	236	17,699	1.33
HF Only	Women	85-89	148	10,473	1.41
HF Only	Women	90+	83	6,103	1.36
HF Only	Men	18-29	20	108,388	0.02
HF Only	Men	30-34	25	40,760	0.06
HF Only	Men	35-39	32	36,359	0.09
HF Only	Men	40-44	83	42,110	0.20
HF Only	Men	45-49	172	44,819	0.38
HF Only	Men	50-54	323	42,508	0.76
HF Only	Men	55-59	356	36,476	0.98
HF Only	Men	60-64	448	32,068	1.40
HF Only	Men	65-69	538	27,327	1.97
HF Only	Men	70-74	530	20,536	2.58
HF Only	Men	75-79	481	16,162	2.98
HF Only	Men	80-84	328	10,555	3.11
HF Only	Men	85-89	117	4,935	2.37
HF Only	Men	90+	33	1,976	1.67
Both: LD + HF	Women	18-29	6	110,408	0.01
Both: LD + HF	Women	30-34	9	42,252	0.02
Both: LD + HF	Women	35-39	17	37,934	0.04
Both: LD + HF	Women	40-44	34	45,989	0.07
Both: LD + HF	Women	45-49	34	48,958	0.07
Both: LD + HF	Women	50-54	62	45,928	0.13
Both: LD + HF	Women	55-59	134	38,388	0.35
Both: LD + HF	Women	60-64	212	34,072	0.62
Both: LD + HF	Women	65-69	338	30,156	1.12
Both: LD + HF	Women	70-74	530	25,812	2.05
Both: LD + HF	Women	75-79	728	23,234	3.13
Both: LD + HF	Women	80-84	750	17,699	4.24
Both: LD + HF	Women	85-89	629	10,473	6.01
Both: LD + HF	Women	90+	476	6,103	7.80
Both: LD + HF	Men	18-29	<6	108,388	0.00
Both: LD + HF	Men	30-34	<6	40,760	0.01
Both: LD + HF	Men	35-39	10	36,359	0.03
Both: LD + HF	Men	40-44	38	42,110	0.09
Both: LD + HF	Men	45-49	81	44,819	0.18
Both: LD + HF	Men	50-54	129	42,508	0.30
Both: LD + HF	Men	55-59	249	36,476	0.68
Both: LD + HF	Men	60-64	371	32,068	1.16
Both: LD + HF	Men	65-69	527	27,327	1.93
Both: LD + HF	Men	70-74	659	20,536	3.21
Both: LD + HF	Men	75-79	727	16,162	4.50
Both: LD + HF	Men	80-84	588	10,555	5.57
Both: LD + HF	Men	85-89	341	4,935	6.91
Both: LD + HF	Men	90+	158	1,976	8.00

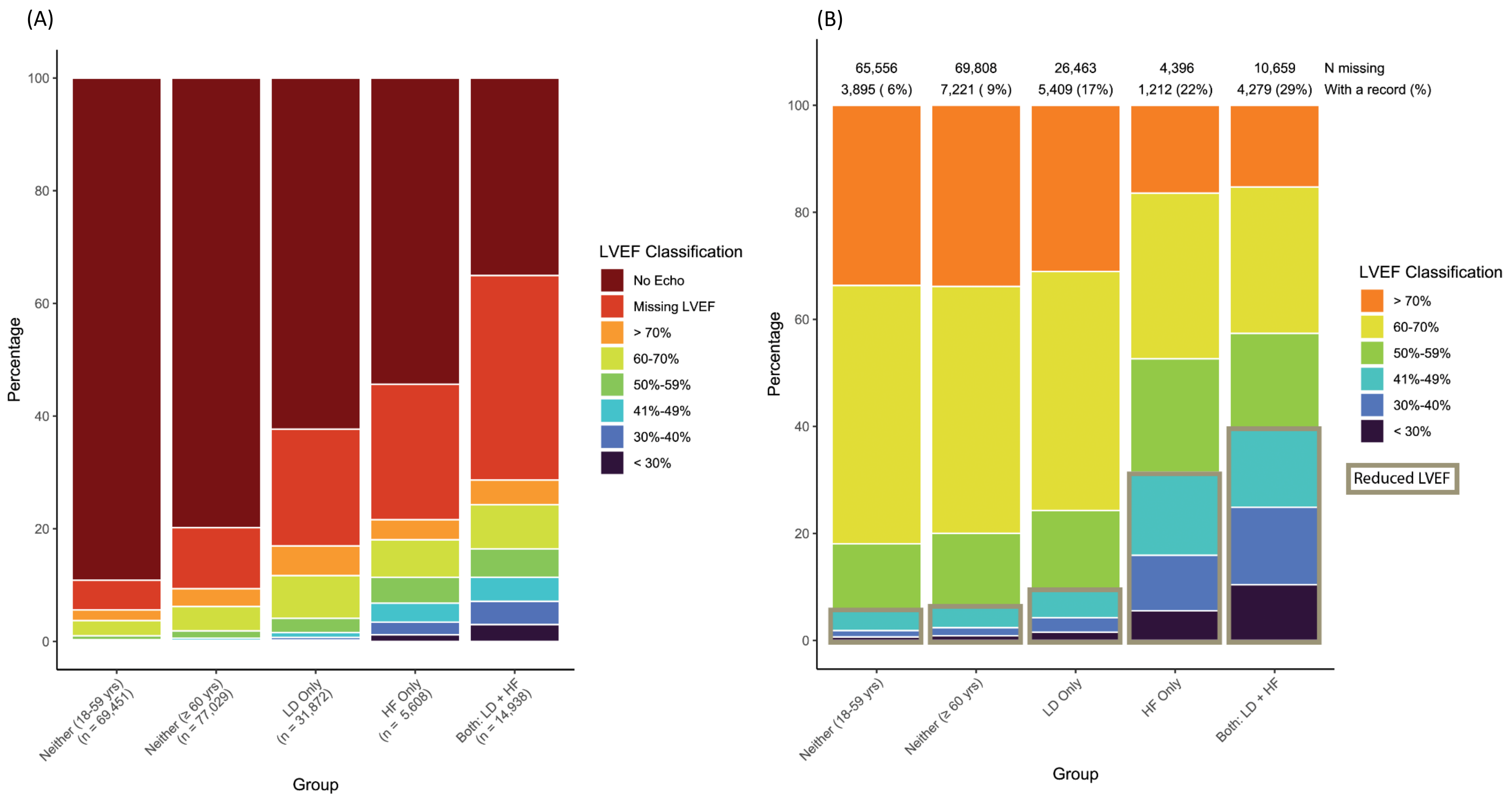

Supplementary Figure 3 Left ventricular ejection fraction (LVEF) data classified by prescription of loop diuretics (LD) and a diagnosis of heart failure (HF)

Data taken from the test closest to the date of the last transition (initiation of loop diuretic or diagnosis of HF) (many bedside echocardiograms would not be included in the electronic health record).

(A) Distribution of missing data and LVEF measurements when available.

(B) Distribution of LVEF only for those with a record. The grey box indicates a reduced ejection fraction (LVEF <50%).

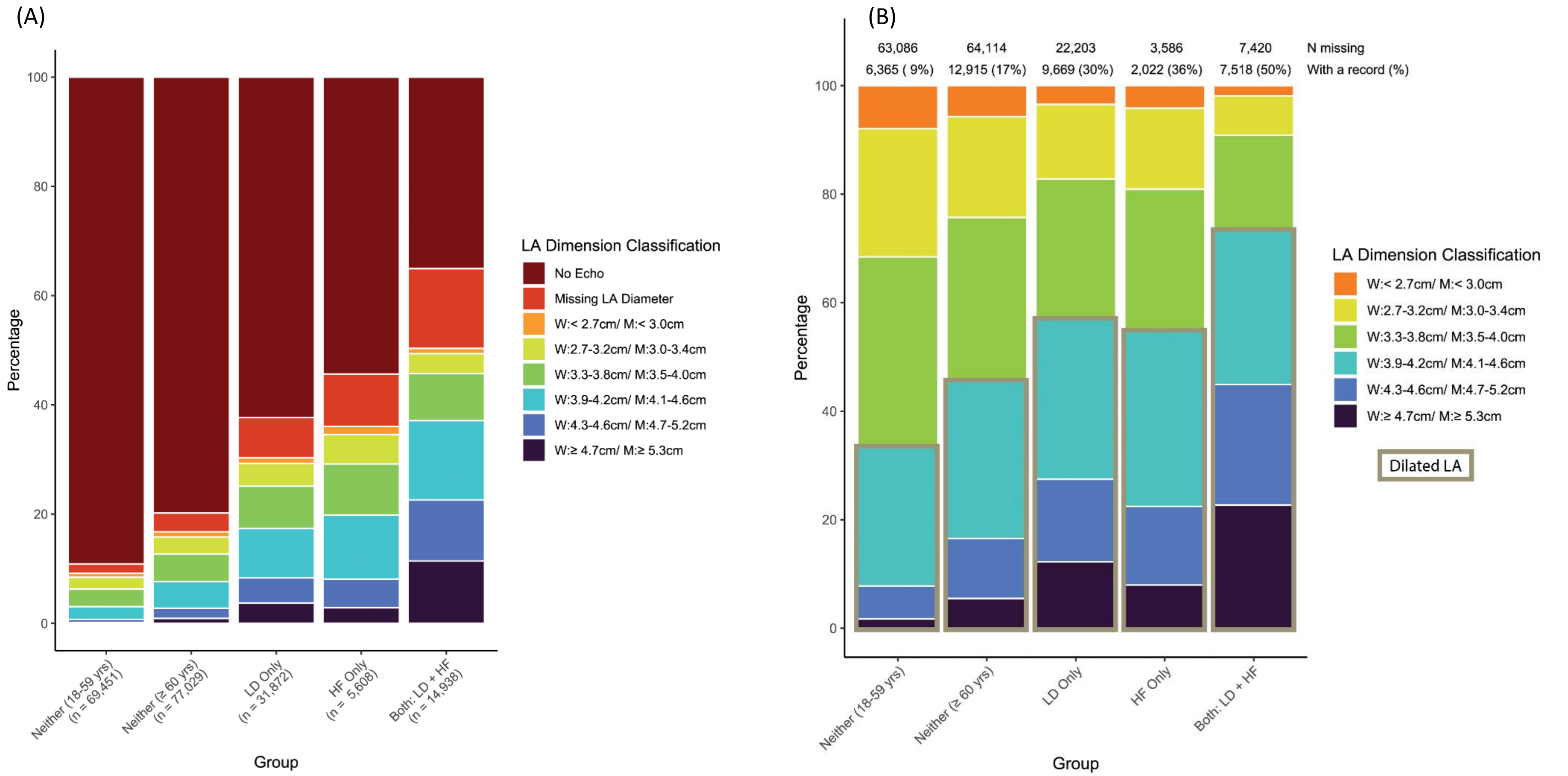

Supplementary Figure 4 Left atrial diameter data classified by prescription of loop diuretics (LD) and a diagnosis of heart failure (HF)

Data was taken from the test closest to the date of the last transition (initiation of loop diuretic or diagnosis of HF) (many bedside echocardiograms would not be included in the electronic health record).

(A) Distribution of missing data and left atrial (LA) measurements when available.

(B) Distribution of left atrial (LA) measurements for those with a record. The grey box indicates a dilated left atrium (LA).

W = women; M = men

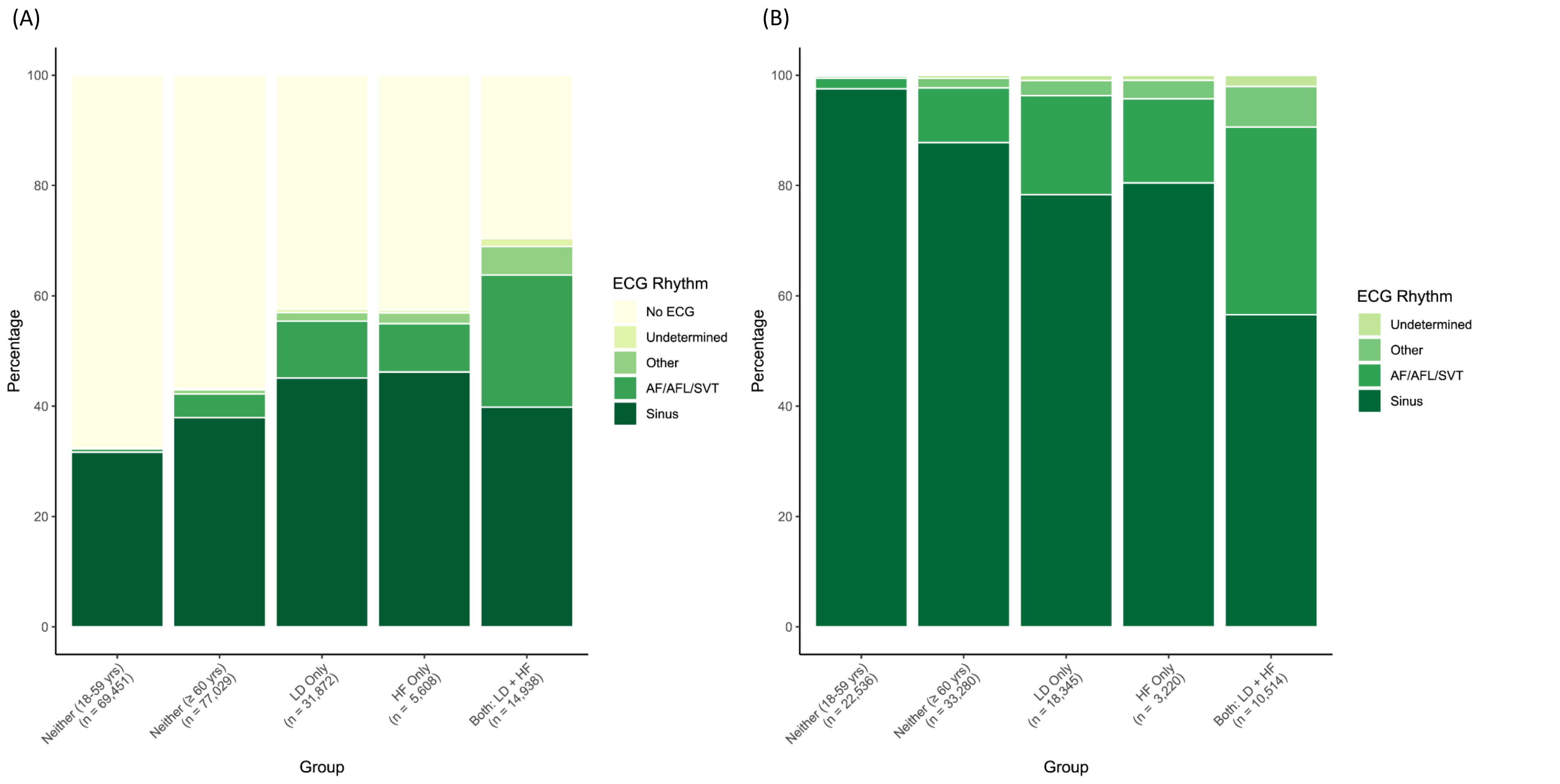

Supplementary Figure 5 Heart rhythm on 12-lead ECG

(A) Rhythm classification by group, including the percentage of patients without a 12-lead ECG, and (B) rhythm classification of those with a recorded ECG. Data and group were taken from the test recorded closest to the date of the last transition from a MUSE-compatible ECG, which may exclude bedside/emergency room tests.

(A) Distribution of missing data and heart rhythm when available.
(B) Distribution of heart rhythm only for those with a record.

Where ECGs were recorded, the 'Neither (18-59 yrs)' group had a median QRS duration (1st – 3rd quartile) of 86 (80-96) ms; 'Neither (18-59 yrs)' had a duration of 88 (80 –98) ms; those dispensed loop diuretics without HF (LD Only) had a duration of 88 (80 – 98) ms; those with a diagnosis of HF without loop diuretics (HF Only) had a duration of 94 (84 – 108) ms; and those with both loop diuretics and HF (Both: LD + HF) had a duration of 98 (86 – 122) ms.

Classification of morbidity and mortality events

Supplementary Table 9 Definitions of disease categories used for classifying causes of death and hospitalisations.

<table>
<tr><th>Causes of death</th><th>Causes of hospitalisation</th></tr>
<tr><td rowspan="2">CV: ICD-10 Chapter IX' Diseases of the circulatory system' (code range I00-I99), except for those classified as an infection.</td><td>Heart Failure: ICD-10 codes: I50 'Heart Failure' (includes I50.0, I50.1, I50.9), I42.0 'Dilated cardiomyopathy', I42.9 'Cardiomyopathy, unspecified', I11.0 'Hypertensive heart disease with (congestive) heart failure', I25.5 'Ischaemic cardiomyopathy', I13.0 'Hypertensive heart and renal disease with (congestive) heart failure', I13.2 'Hypertensive heart and renal disease with both (congestive) heart failure and renal failure'.</td></tr>
<tr><td>Other Cardiovascular Disorders: ICD-10 Chapter IX 'Disorders of the circulatory system' (I00 - I99), excluding codes related to heart failure or infections.</td></tr>
<tr><td colspan="2">Neoplasms: ICD-10 Chapter II 'Neoplasms' (C00 - D48)</td></tr>
<tr><td colspan="2">Infections: Individual codes are listed in Table Supplementary Table 10.</td></tr>
<tr><td rowspan="8">Other: any code not falling into any of the above categories, including the 257 deaths where the cause of death was unknown.</td><td>Chronic Respiratory Disease: individual codes listed in Supplementary Table 11.</td></tr>
<tr><td>Digestive Diseases: ICD-10 Chapter XI 'Diseases of the digestive system' (K00-K93), excluding codes categorised as infections.</td></tr>
<tr><td>Eye & Adnexa: ICD-10 Chapter VII 'Disease of the eye and adnexa' (H00-H59), excluding codes categorised as infections.</td></tr>
<tr><td>Injury: ICD-10 Chapter XIX 'Injury, poisoning and certain other consequences of external causes' (S00-T98), and ICD-10 Chapter XX 'External Causes of morbidity and mortality' (V01-Y09).</td></tr>
<tr><td>Mental Health & Neurological Disorders: ICD-10 Chapter IV 'Diseases of the nervous system' (G00-G99), excepting selected codes categorised as infections and ICD-10 Chapter V 'Mental and behavioural disorders' (F00-F99).</td></tr>
<tr><td>Musculoskeletal Disorders: ICD-10 Chapter XIII 'Diseases of the musculoskeletal system and connective tissue'.</td></tr>
<tr><td>Renal: ICD-10 subchapters 'Renal failure' (N17-N19), 'Glomerular disease' (N00-N08), 'Renal tubule-interstitial disease' (N10-N16), and 'Other disorders of kidney and ureter' (N25-N29), excluding codes classified as infections.</td></tr>
<tr><td>Other: ICD-10 Chapter XVIII 'Symptoms, signs, and abnormal clinical laboratory findings, not elsewhere classified' (R00-R99), as well as any code not falling into the above categories.</td></tr>
</table>

Supplementary Table 10 Codes used to define infection admissions in SMR01

Unless explicitly noted otherwise, three- and four-digit ICD-10 codes contain all codes below them.

Infection classification	ICD-10 Code	Code Description
Infectious disease	A00 - B99	Certain infectious and parasitic disease
Other respiratory infections	H65	Nonsuppurative otitis media
	H65.0	Acute serous otitis media
	H65.1	Other acute nonsuppurative otitis media
	H65.2	Chronic serous otitis media
	H65.3	Chronic mucoid otitis media
	H65.4	Other chronic nonsuppurative otitis media
	H66.0	Acute suppurative otitis media
	H66.1	Chronic tubotympanic suppurative otitis media
	H66.2	Chronic atticoantral suppurative otitis media
	H72	Perforation of tympanic membrane
	H73	Other disorders of tympanic membrane
	H80	Otosclerosis
	H83	Other diseases of inner ear
	J01	Acute sinusitis
	J02	Acute pharyngitis
	J03	Acute tonsillitis
	J04	Acute laryngitis and tracheitis
	J05	cute obstructive laryngitis [croup] and epiglottitis
	J06	Acute upper respiratory infections of multiple and unspecified sites
	J09	Influenza due to identified zoonotic or pandemic influenza virus
	J10	Influenza due to identified seasonal influenza virus
	J11	Influenza, virus not identified
	J12	Viral pneumonia, not elsewhere classified
	J13	Pneumonia due to Streptococcus pneumoniae
	J14	Pneumonia due to Haemophilus influenzae
	J15	Bacterial pneumonia, not elsewhere classified
	J16	Pneumonia due to other infectious organisms, not elsewhere classified
	J17	Pneumonia in diseases classified elsewhere
	J18	Pneumonia, organism unspecified
	J20	Acute bronchitis
	J21	Acute bronchiolitis
	J22	Unspecified acute lower respiratory infection
	J32.9	Chronic sinusitis, unspecified
	J40	Bronchitis, not specified as acute or chronic
	J85.1	Abscess of lung with pneumonia
	J86.9	Pyothorax without fistula
	K67.3	Tuberculous peritonitis
	K93.0	Tuberculous disorders of intestines, peritoneum and mesenteric glands
	N74.1	Female tuberculous pelvic inflammatory disease
UTI	N11.0	Nonobstructive reflux-associated chronic pyelonephritis
	N11.8	Other chronic tubulo-interstitial nephritis
	N15.0	Balkan nephropathy
	N15.1	Renal and perinephric abscess
	N30	Cystitis
	N34	Irradiation cystitis
	N39.0	Cystitis, unspecified
	G00	Bacterial meningitis, not elsewhere classified

Other infectious diseases	G03	Meningitis due to other and unspecified causes
	G04	Encephalitis, myelitis and encephalomyelitis
	H70.1	Chronic mastoiditis
	I00	Rheumatic fever without mention of heart involvement
	I01	Rheumatic fever with heart involvement
	I02	Rheumatic chorea
	I30	Acute pericarditis
	I33	Acute and subacute endocarditis
	I40	Acute myocarditis
	L03	Cellulitis

Supplementary Table 11 Codes used to define chronic respiratory disease admissions in SMR01

Unless explicitly noted otherwise, three- and four-digit ICD-10 codes contain all codes below them.

ICD-10 code	Code Description
D86.0	Sarcoidosis of lung
D86.1	Sarcoidosis of lymph nodes
D86.2	Sarcoidosis of lung with sarcoidosis of lymph nodes
D86.8	Sarcoidosis of other and combined sites
D86.9	Sarcoidosis, unspecified
J38.0	Paralysis of vocal cords and larynx
J38.6	Stenosis of larynx
J39.0	Retropharyngeal and parapharyngeal abscess
J39.2	Other diseases of pharynx
J39.8	Other specified diseases of upper respiratory tract
J41	Simple and mucopurulent chronic bronchitis
J42	Unspecified chronic bronchitis
J43	Emphysema
J44	Other chronic obstructive pulmonary disease
J45	Asthma
J46	Status asthmaticus
J47	Bronchiectasis
J60	Coal worker pneumoconiosis
J61	Pneumoconiosis due to asbestos and other mineral fibres
J63	Pneumoconiosis due to other inorganic dusts
J64	Unspecified pneumoconiosis
J67.0	Farmer lung
J67.9	Hypersensitivity pneumonitis due to unspecified organic dust
J69.0	Pneumonitis due to food and vomit
J81	Pulmonary oedema
J82	Pulmonary eosinophilia, not elsewhere classified
J84	Other interstitial pulmonary diseases
J90	Pleural effusion, not elsewhere classified
J92.9	Pleural plaque with presence of asbestos
J93.1	Other spontaneous pneumothorax
J93.9	Pneumothorax, unspecified
J94.1	Fibrothorax
J94.8	Other specified pleural conditions
J96.1	Chronic respiratory failure
J96.9	Respiratory failure, unspecified
J98.1	Pulmonary collapse
J98.4	Other disorders of lung
J98.8	Other specified respiratory disorders
J98.9	Respiratory disorder, unspecified

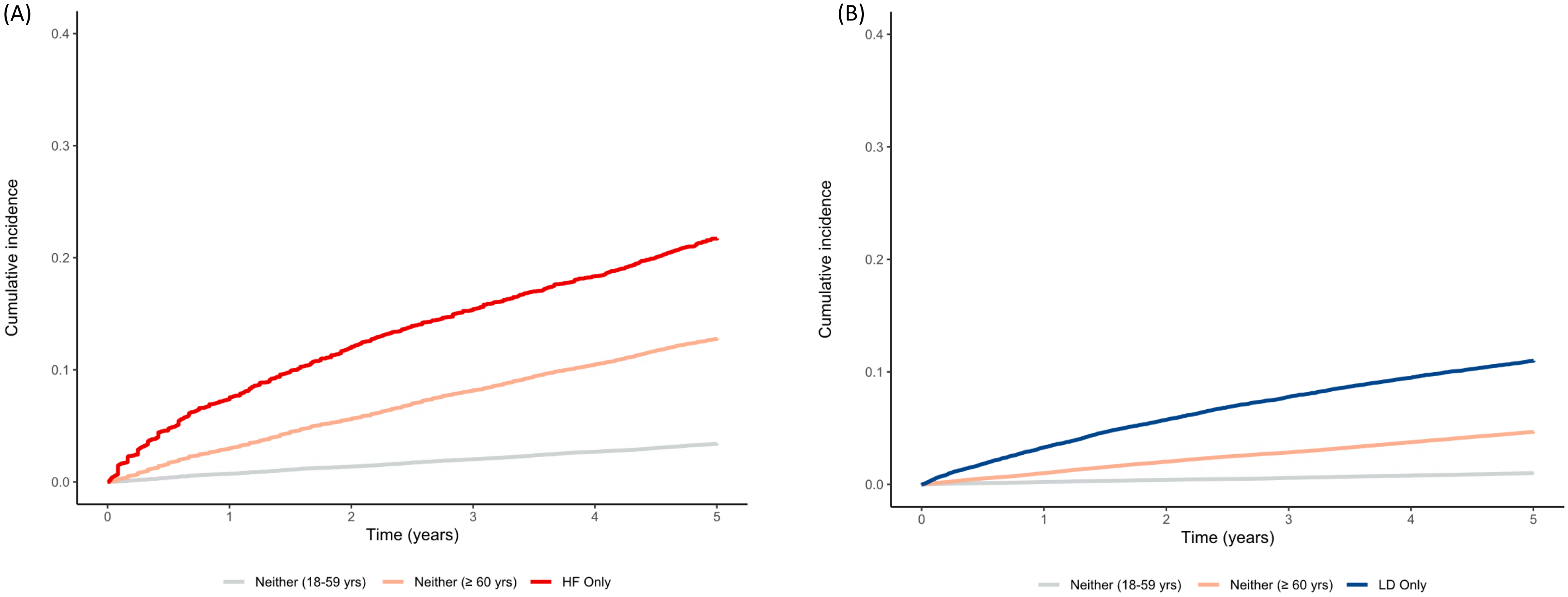

Number at risk (A)

Neither (18-59 yrs)	72236	71100	69989	68786	67494	65758
Neither (≥ 60 yrs)	89699	83908	78551	73335	68379	63547
HF Only	5156	4536	4121	3778	3505	3205

Number at risk (B)

Neither (18-59 yrs)	72236	71440	70621	69690	68676	67219
Neither (≥ 60 yrs)	89699	85443	81185	77049	72789	68484
LD Only	23963	20990	18558	16420	14585	13009

Supplementary Figure 6 5-year cumulative incidence of loop diuretic (LD) initiation or diagnosis of heart failure (HF)

(A) Cumulative incidence of the initiation of loop diuretics in the three relevant populations who had not received a loop diuretic at baseline (Neither [18-59 yrs], Neither [≥ 60 yrs], and HF Only).

(B) Cumulative incidence of the diagnosis of HF in the three relevant populations without a diagnosis at baseline (Neither [18-59 yrs], Neither [≥ 60 yrs], and loop diuretic [LD] Only).

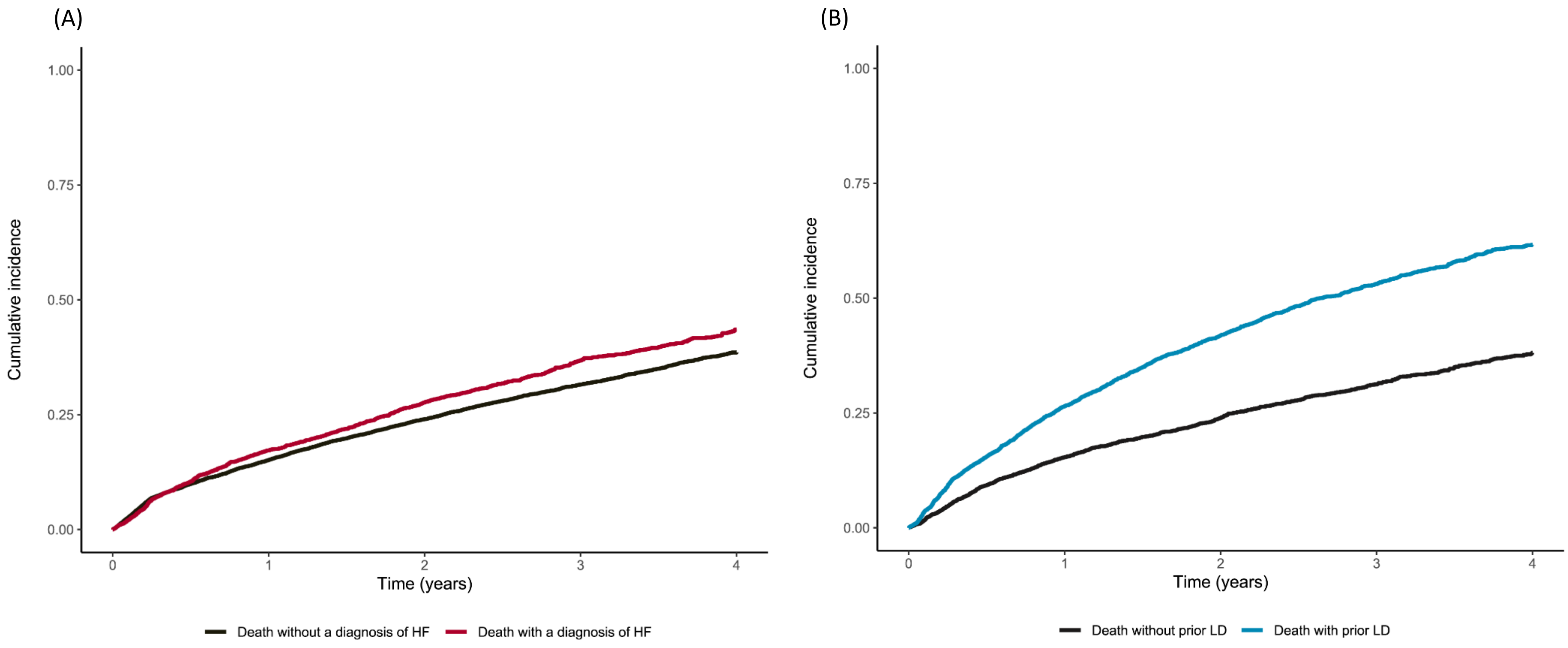

Number at risk (A)

Death without a diagnosis of HF	11906	8320	5763	3597	1745
Death with a diagnosis of HF	3097	2072	1443	877	432

Number at risk (B)

Death without prior LD	3534	2491	1767	1192	574
Death with prior LD	4012	2407	1449	820	368

Supplementary Figure 7: 4-year mortality from initiation of loop diuretics (LD) or diagnosis of heart failure (HF) during follow-up

(A) For patients initially in the 'neither' group, all-cause mortality from the initiation of loop diuretics in those with and without a prior diagnosis of heart failure (when the diagnosis of HF and initiation of loop diuretics were concurrent, HF was considered the prior event).

(B) For patients initially in the 'neither' group, all-cause mortality from the diagnosis of heart failure in those who had or had not been initiated on loop diuretics (when the diagnosis of HF and initiation of loop diuretics were concurrent, loop diuretic initiation was considered the prior event).

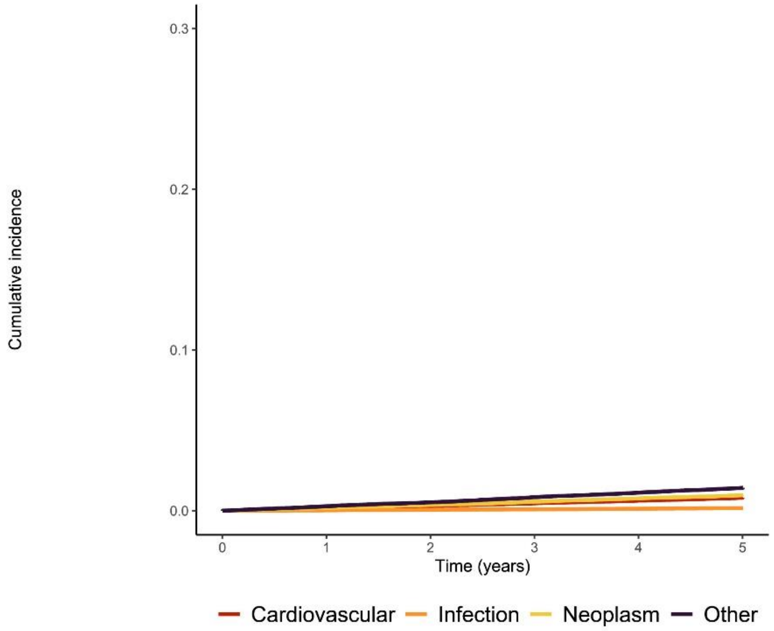

Number at risk

Neither (18-59 yrs)	72236	71588	70892	70085	69194	67870

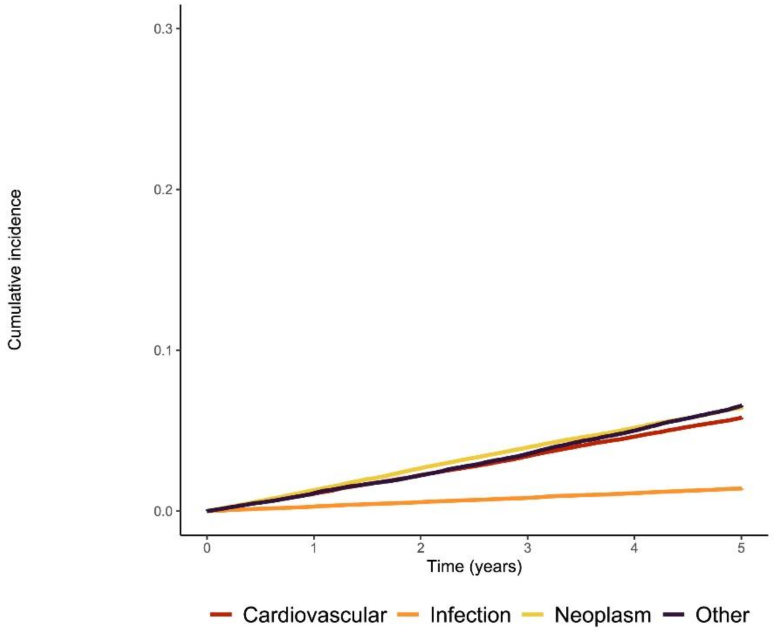

Number at risk

Neither (≥ 60 yrs)	89699	86244	82681	78999	75195	71292

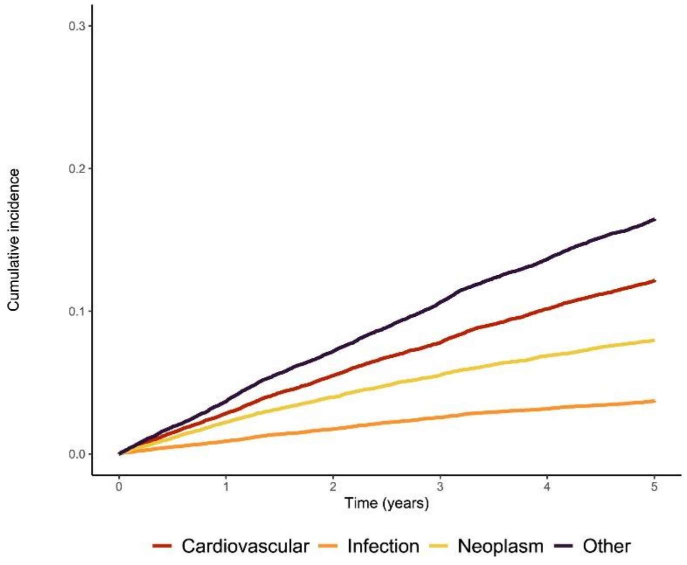

Number at risk

LD Only	23963	21644	19536	17591	15822	14266

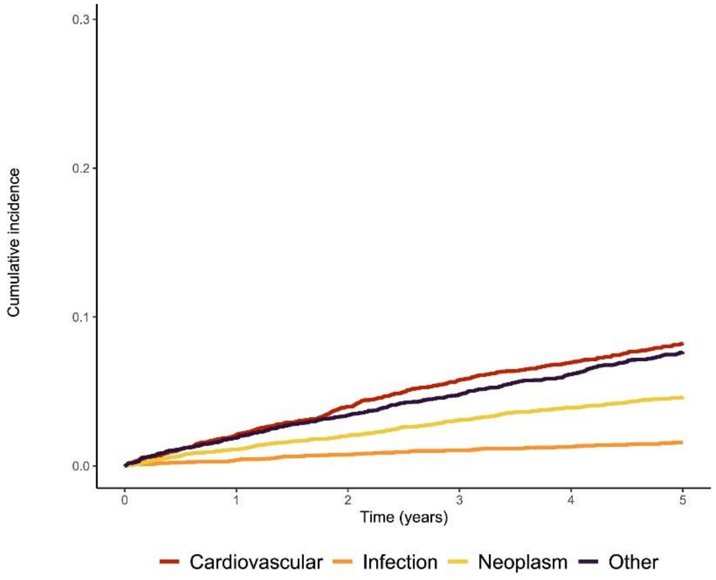

Number at risk

HF Only	5156	4875	4631	4394	4204	3998

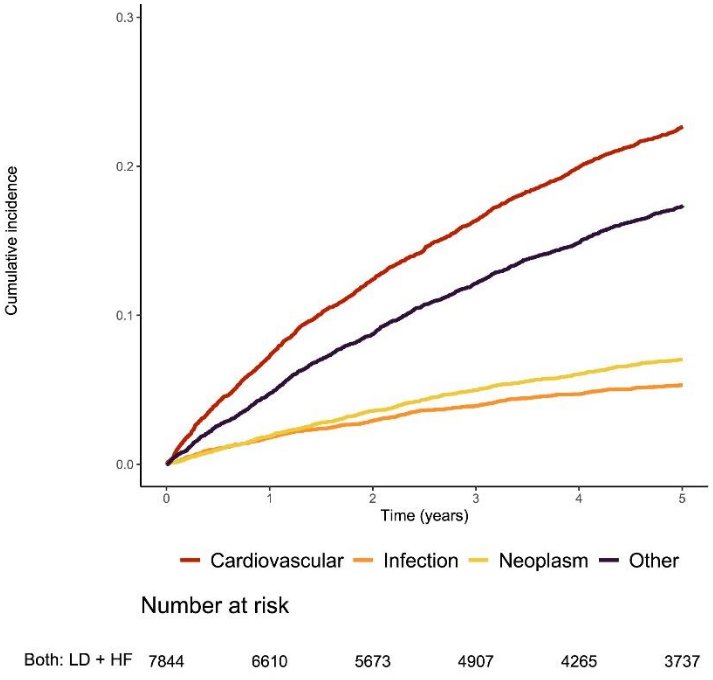

Supplementary Figure 8 5-year cumulative incidence of causes of death by baseline group classification on 31 December 2011.

Data on the loop diuretic only population

Supplementary Table 12 Demographics and co-morbidities of individuals with loop diuretics only at baseline classified by survival status at the end of follow-up

	Censored n = 14,322	Died n = 9,641
Age at baseline (years)	71 (61 - 78)	81 (74 - 87)
Length of follow-up (years)	5.00 (5.00 - 5.00)	2.19 (1.04 - 3.43)
Sex		
Women	10,581 (74%)	6,194 (64%)
Men	3,741 (26%)	3,447 (36%)
Ethnicity		
White	12,244 (85%)	8,416 (87%)
Missing	1,803 (13%)	1,099 (11%)
Other	275 (2%)	126 (1%)
Scottish Index of Multiple Deprivation		
1 (most deprived)	6,355 (44%)	4,170 (43%)
2	2,718 (19%)	1,806 (19%)
3	1,808 (13%)	1,428 (15%)
4	1,559 (11%)	1,077 (11%)
5 (least deprived)	1,882 (13%)	1,160 (12%)
Co-morbidities*		
History of hypertension	5,884 (41%)	3,226 (33%)
Diabetes mellitus	3,185 (22%)	1,673 (17%)
Thyroid disease	572 (4%)	511 (5%)
CAD (including myocardial infarction)	4,396 (31%)	(50%)
Myocardial Infarction	1,302 (9%)	1,183 (12%)
Valve disease	565 (4%)	574 (6%)
Atrial fibrillation/ flutter	1,820 (13%)	2,073 (22%)
Peripheral arterial disease	421 (3%)	502 (5%)
Stroke	1,140 (8%)	1,245 (13%)
Chronic obstructive pulmonary disease	2,533 (18%)	2,083 (22%)
Liver disease⊕	169 (1%)	216 (2%)
Cancer	1,029 (7%)	1,251 (13%)
Dementia	257 (2%)	938 (10%)

Data are frequencies (%) for categorical values or median (1st – 3rd quartile) for continuous values.
* History of a coded record on or before 1st January 2012
⊕ Defined by the presence of liver fibrosis, sclerosis, or cirrhosis
CAD, coronary artery disease

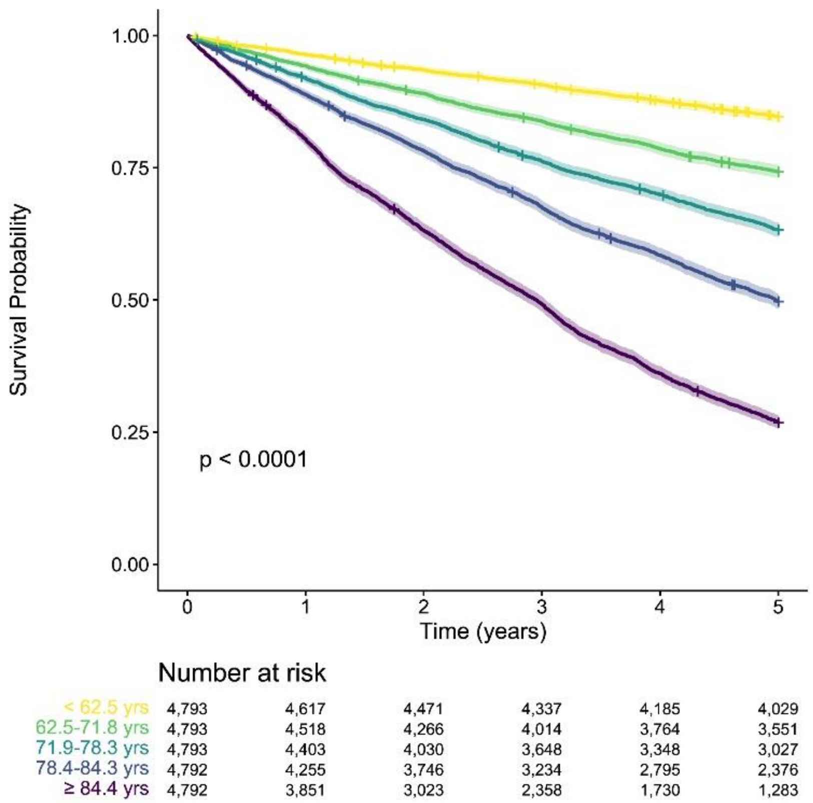

Supplementary Figure 9 5-year Kaplan-Meier curves to compare survival rates of those with only a loop diuretic at baseline by quintile of age as of 1 January 2012.

Time-dependent covariate analysis

Time-dependent analysis was conducted where the diagnosis of heart failure and the initiation of a loop diuretic were triggers for updating group and comorbidity status.

If the calculations are conducted using the time-dependent groups, that same patient contributed no admissions and 367 days (or 1.00 admission-free patient-years) of admission-free follow-up to the Neither (≥ 60 yrs) group. Then, the patient contributed one infection admission, one injury admission, and 726 admission-free days (or 1.99 admission-free patient-years) of follow-up to where they had only a loop diuretic (LD only group). Finally, the patient changed groups for the second time based on the heart failure diagnosis in hospital. From this point, the patient contributed one heart failure admission, one infection admission, and one other CV admission plus 725 admission-free days (or 1.98 admission-free patient-years) of follow-up to where they had were on both loop diuretics and diagnosed with heart failure (Both: LD + HF) before dying on 31st December 2016. In total, the patient contributed 367 days to 'Neither (≥ 60

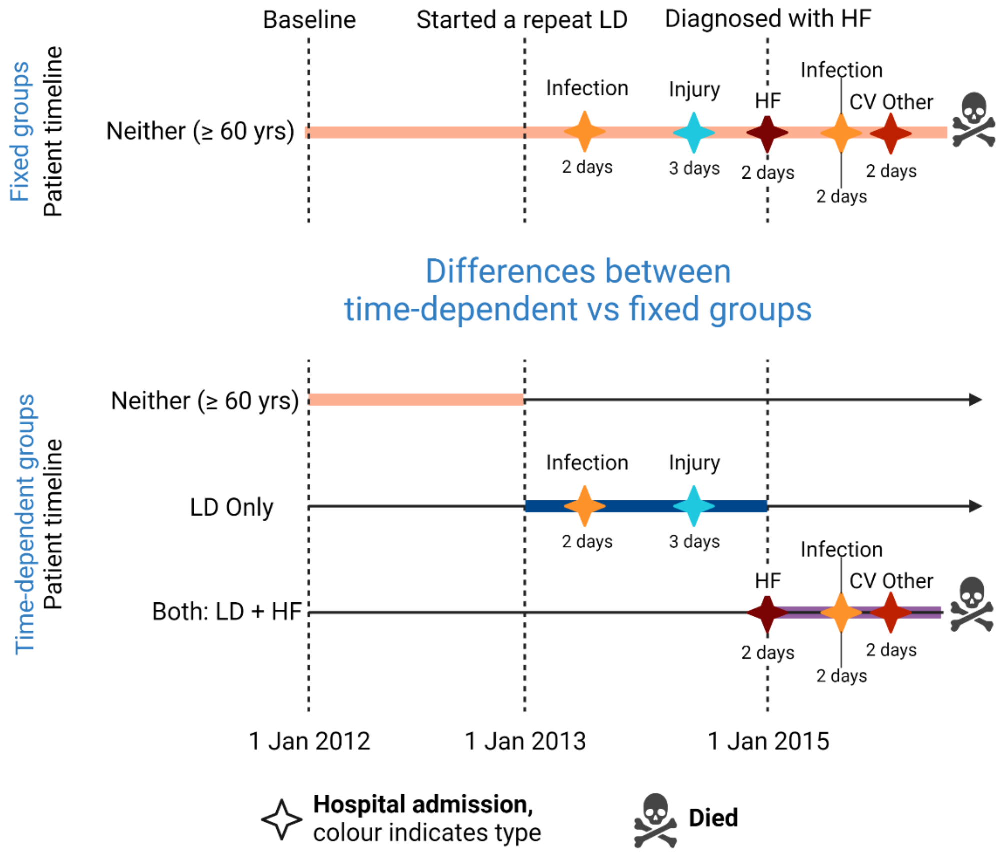

Supplementary Figure 10 Explanation of changes in assigning patient time at risk based on the analysis method.

LD, loop diuretic; HF, heart failure

(top) Patient time at risk was calculated using baseline group classification, while (bottom) patient time at risk was calculated using time-dependent groups based on the presence or absence of LD and HF. If calculations are conducted using the fixed baseline group, the patient's follow-up started on 1 January 2012 and ended when the patient died on the 31 of December 2016. Subtracting the 11 days the patient was admitted to hospital, the patient contributed two infection admissions, one injury admission, one heart failure admission, and one other CV admission, plus 1,816 days or 4.97 patient-years of follow-up to the Neither (≥ 60 yrs) group admissions calculations. Regarding mortality, the patient contributed 1,827 days to the Neither (≥ 60 yrs) group and the death is also attributed to the Neither (≥ 60 yrs).

If calculations are conducted using the time-dependent groups, the patient contributed 365 days to the Neither (≥ 60 yrs) group, and 731 days to each of LD only and Both: LD + HF, and the death record is attributed to the Both: LD + HF group. Where the diagnosis of heart failure and the initiation of loop diuretic were recorded on the same day, patients moved directly having both loop diuretics and HF (Both: LD + HF) without first contributing to either loop diuretic only or HF only.

Handling missing eGFR data

Based on previous analysis using West of Scotland Safe Haven Data, the absence of blood tests is associated with lower hazards of all-cause mortality(24); therefore, missing eGFR tests were classified as missing not at random. The impact of the dependence of the analysis on the missing values was measured by adding eGFR, where available, as a penalised spline with 5 knots (4 degrees of freedom) and, where applicable, substituting the missing values sequentially with a credible range of values(25). Five analyses were run based on the following:

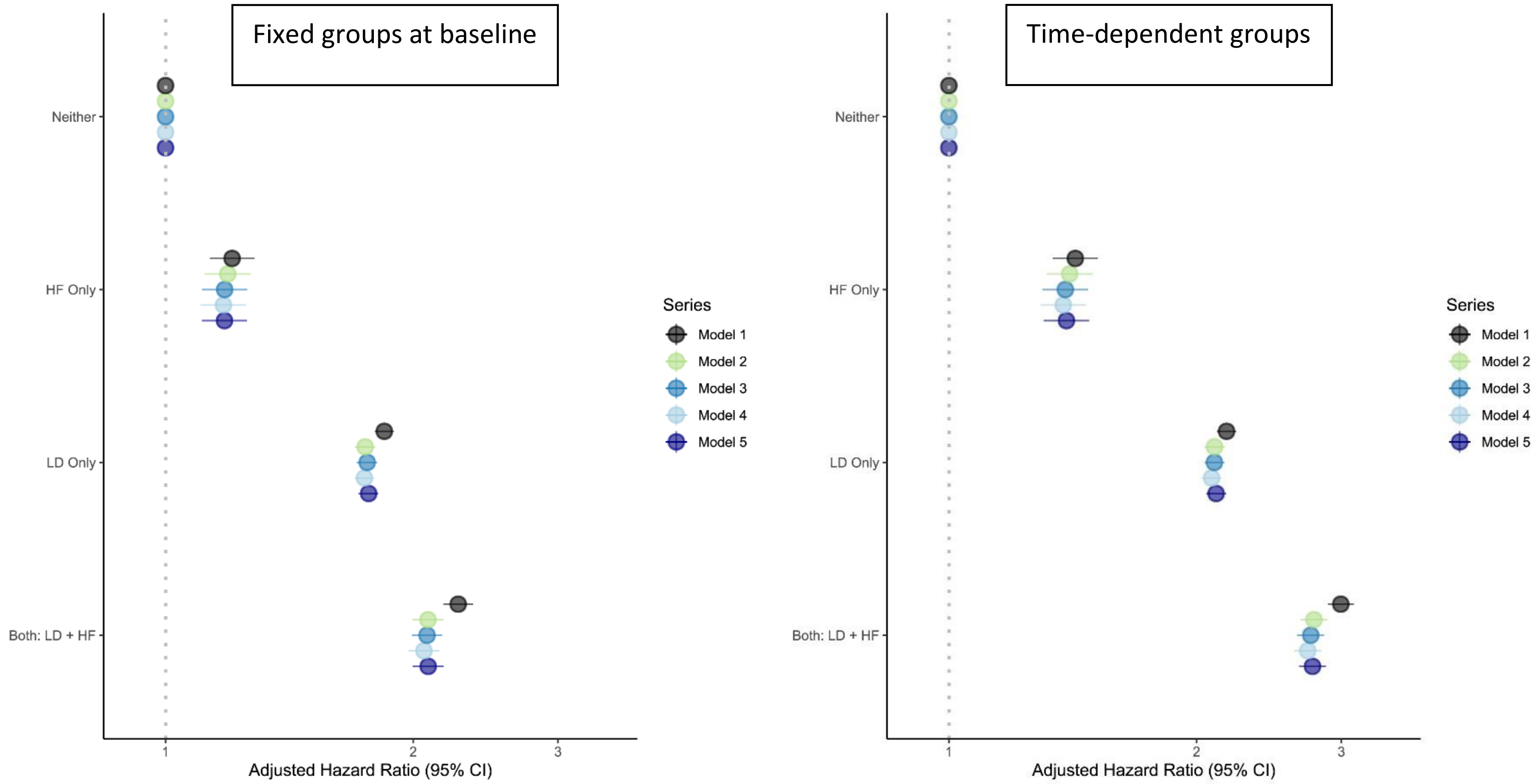

Supplementary Figure 11 Changes in 5-year all-cause mortality adjusted hazard ratios based on using complete eGFR case analysis against replacing missing values in turn with available per group median value, first quartile per group value, and third quartile per group value.

Model 1) adjusting for age as a continuous variable, sex, Scottish Index of Multiple Deprivation, and comorbidities (base model). Model 2) Adjusting for the covariates used in Model 1 plus eGFR for those with an eGFR measurement in the prior two years; Model 3) Adjusting for the covariates used in Model 1 plus eGFR, substituting the missing eGFR values with the per group median value. Model 4) Adjusting for the covariates used in Model 1 plus eGFR, substituting the missing values with the first quartile value per group. Finally, Model 5) Adjusting for the covariates used in Model 1 plus eGFR, substituting the missing values with the third quartile value per group.

Supplementary Table 12 Median (1st-3rd quartile) values of eGFR at baseline and time-dependent status for the study population classified by prescription of loop diuretics (LD) and diagnosis of heart failure (HF)

Baseline	Neither (18-59 yrs)	Neither (≥ 60 yrs)	LD Only	HF Only	Both: LD + HF
n	72,236	89,699	23,963	5,156	7,844
Age (years)	50 (41 - 55)	72 (66 - 78)	75 (65 - 83)	69 (59 - 78)	77 (68 - 83)
Patient-record of eGFR	57,305 (79%)	83,815 (93%)	22,575 (94%)	4,962 (96%)	7,656 (98%)
eGFR (mL/min/1.73m2)	99 (90 - 106)	78 (64 - 88)	71 (52 - 85)	81 (64 - 92)	61 (43 - 79)
Time-dependent covariates					
n	72,236	89,699	35,869	8,690	14,938
Time-dependent age (years)	50 (41 - 55)	72 (66 - 78)	75 (65 - 83)	72 (62 - 80)	78 (70 - 84)
Patient record of eGFR in prior two years	57,305 (79%)	83,815 (93%)	34,262 (96%)	8,463 (97%)	14,728 (99%)
eGFR (mL/min/1.73m2)	99 (90– 106)	78 (64 – 88)	71 (53 – 85)	77 (58 – 90)	61 (42 – 78)

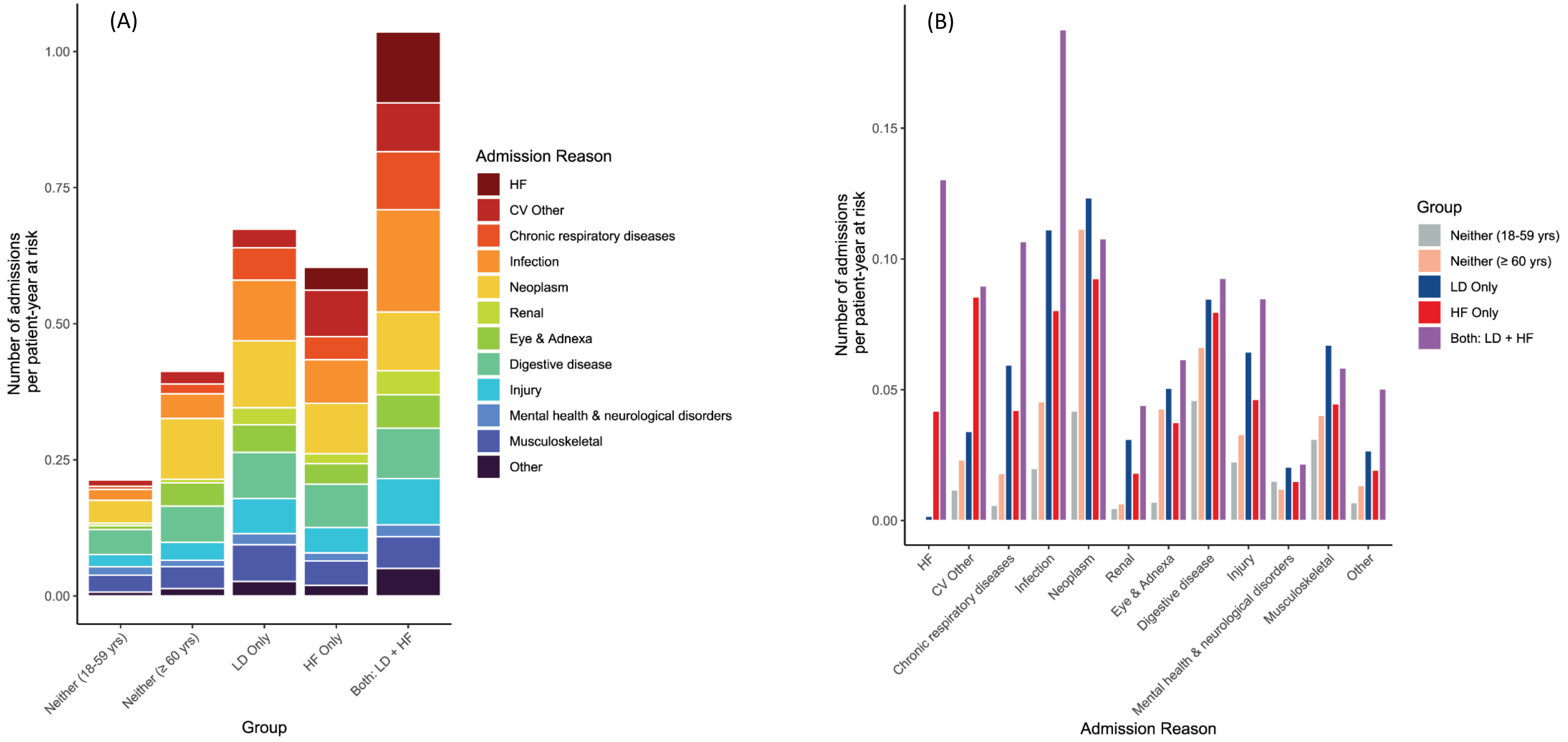

Supplementary Figure 12 Number and type of admissions per person-year at risk from 1 January 2012 through 31 December 2016, where the group is a time-dependent covariate.

(A) Number of admissions per person-year at risk reported by group spit by admission reason. The number of admissions is split by admission reason.

(B) Number of admissions per person-year at risk reported by admission reason, split by group.

LD, loop diuretic; HF, heart failure

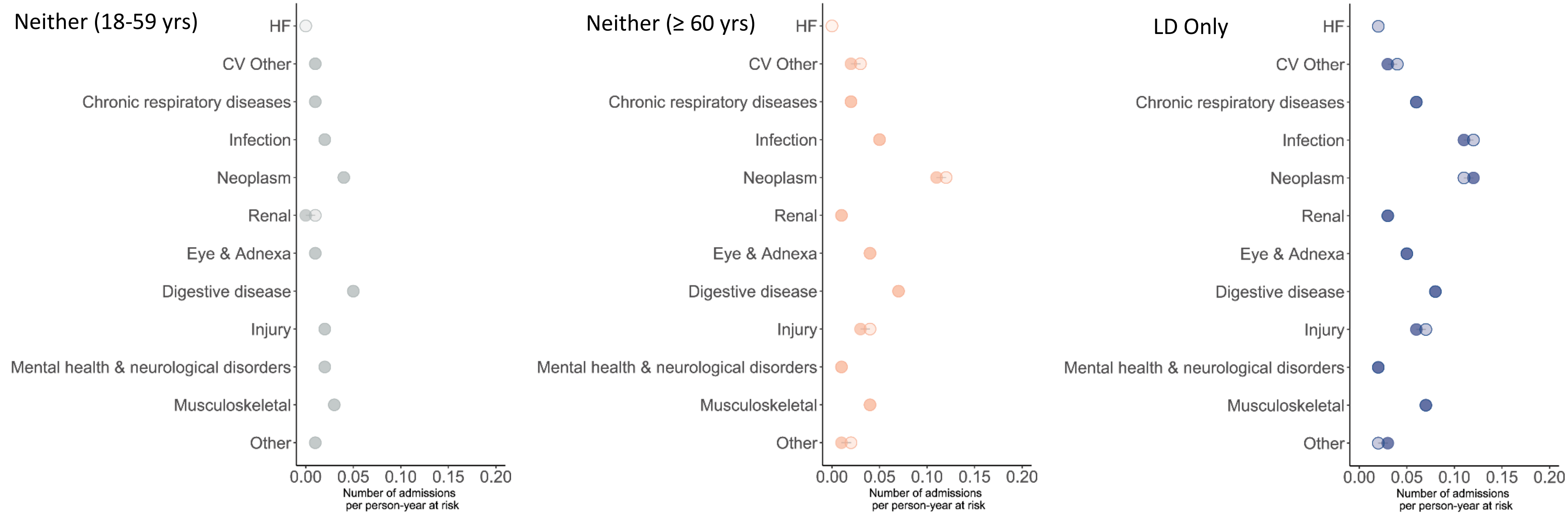

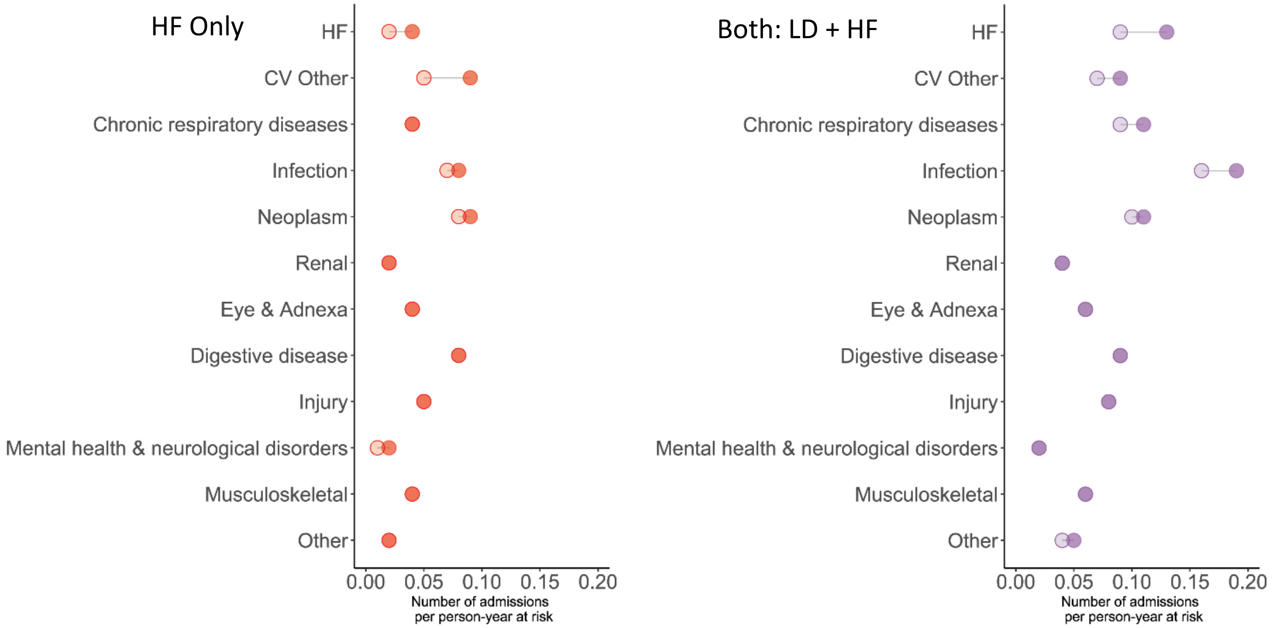

Supplementary Figure 13 Admission rates using baseline (light circle) versus time-dependent (dark circle)

If circles overlap entirely, only the time-dependent changes in the classification circle are visible.

While baseline classification attributes all admissions to the patient’s baseline group, the time-dependent classification only attributes admissions and time at risk accrued while classified as a specific group (i.e., a ‘neither’ patient contributes time and events to the LD only at the initiation of LD and may contribute time and events to Both: LD + HF upon receiving a diagnosis of HF (see above for further details).

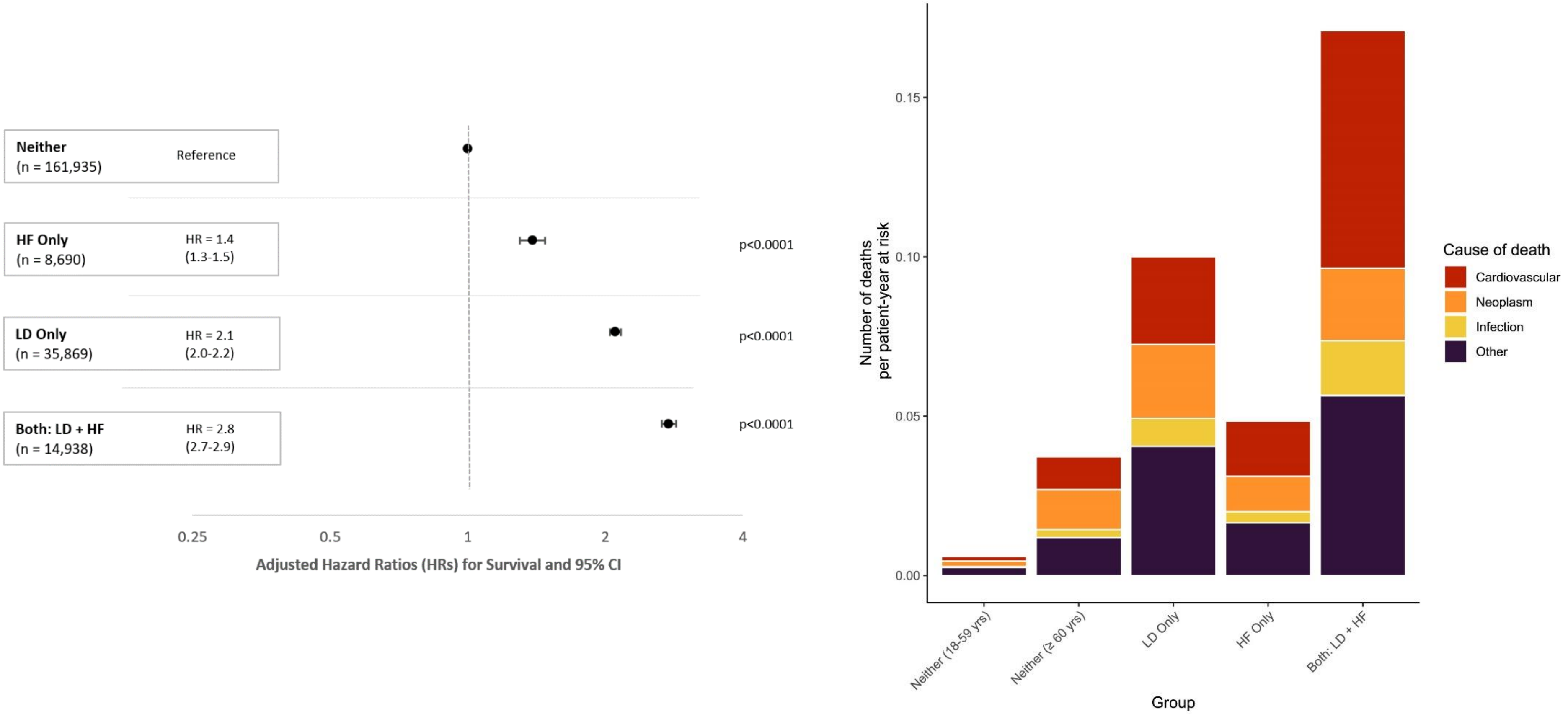

Supplementary Figure 14 5-year mortality using time-dependent groups.

(left) Forest plot of hazard ratios (HR) with 95% confidence intervals (CI) for all-cause mortality by time-dependent group between 1 January 2012 through 31 December 2016. The model was adjusted for by age, sex, SIMD, time-dependent comorbidity status, and closest eGFR in the prior two years.

(right) Number of deaths per patient-year at risk by group, stratified by cause of death.

LD, loop diuretic; HF, heart failure

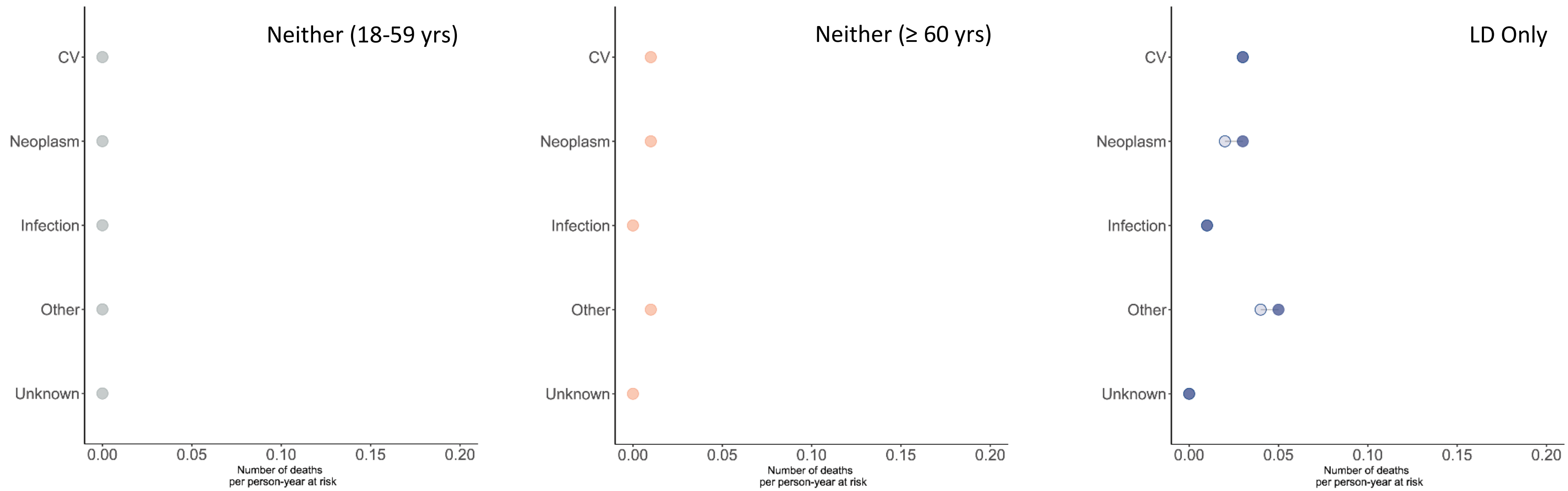

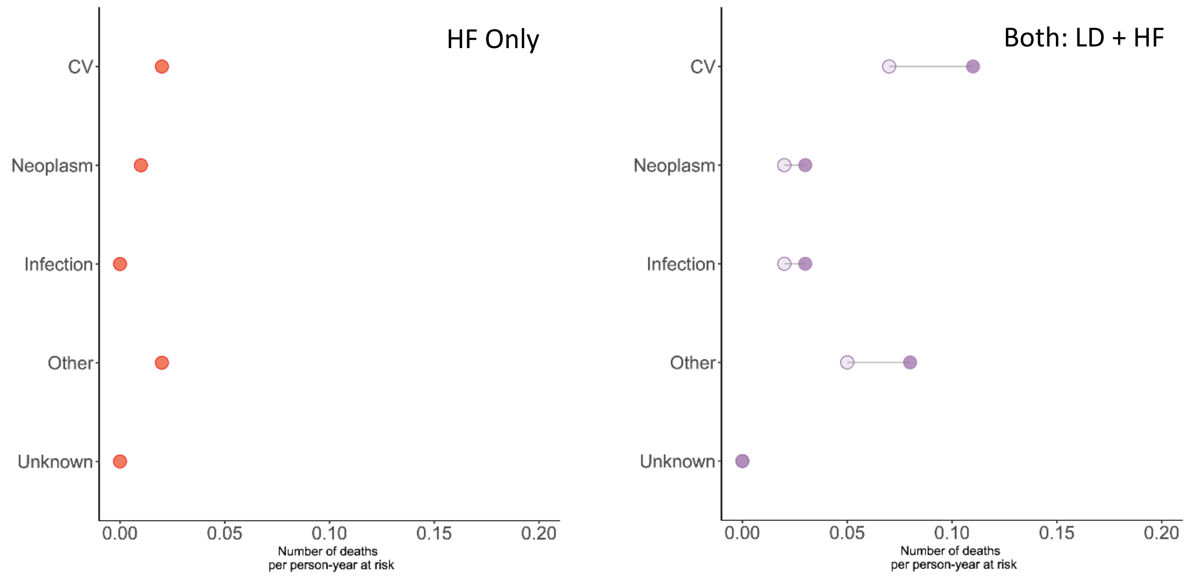

Supplementary Figure 15 Mortality rates using baseline (light circle) versus time-dependent (dark circle) classifications.

If circles overlap entirely, only the time-dependent results can be seen.

With time-dependent classification, deaths and time at risk were attributed to 'LD Only' where the patient either only had a LD at baseline and was never labelled as having HF, or only the time period following the initiation of a LD during follow-up and did not receive a diagnosis of HF. Under time-dependent classification, patients in Both: LD + HF fall under one of the following scenarios: a) classified as Both: LD + HF at baseline; b) started on a LD only and were subsequently diagnosed with HF; c) started as diagnosed with HF only and were subsequently initiated on a repeat LD; or d) in a 'neither' group and subsequently diagnosed with heart failure and initiated on a LD in any order. Only the time and events which occur after an LD has been initiated and heart failure diagnosed count towards Both: LD + HF.

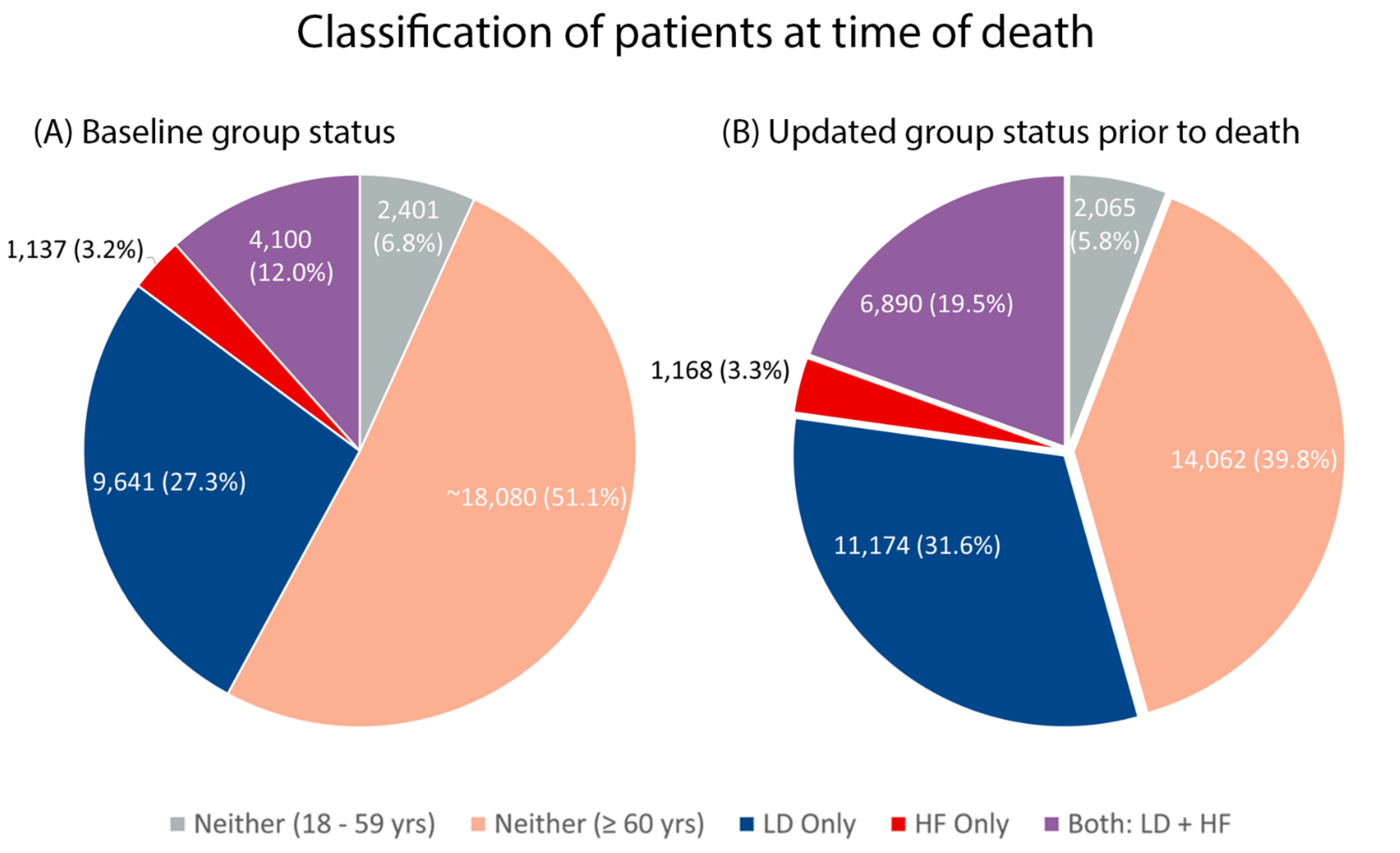

Supplementary Figure 16: Classification of group status at time of death using (A) baseline and (B) time-dependent classification prior to death during 5 years of follow-up.

References

1. NHS Digital. DCB0084: OPCS Classification of Interventions and Procedures 2019 [updated 4 December 2019 9:13 am. Available from: https://digital.nhs.uk/data-and-information/information-standards/information-standards-and-data-collections-including-extractions/publications-and-notifications/standards-and-collections/dcb0084-opcs-classification-of-interventions-and-procedures.

2. Booth N. What are the Read Codes? In: Classification CfCa, editor. Health Libraries Review. Woodgate, Loughborough, Leics LE11 2TG1994. p. 177-82.

3. Public Health Scotland. PTI Statistics, Grouping of codes for conditions (RCGs) 2020 [updated 16 March 2022; cited 2023. Available from: https://www.isdscotland.org/Health-Topics/General-Practice/GP-Consultations/Grouping-clinical-codes.asp#:~:text=Read%20codes%20(version%202%2C%20Scottish,and%20Social%20Care%20Information%20website.

4. Public Health Scotland. SMR Completeness 2020 [Available from: https://www.isdscotland.org/Products-and-Services/Data-Support-and-Monitoring/SMR-Completeness/.

5. ISD Scotland. SMR01 - Summary of Rules [Available from: https://www.ndc.scot.nhs.uk/Dictionary-A-Z/Definitions/index.asp?Search=S&ID=997&Title=SMR01%20-%20Summary%20of%20Rules.

6. Conrad N, Judge A, Tran J, Mohseni H, Hedgecott D, Crespillo AP, et al. Temporal trends and patterns in heart failure incidence: a population-based study of 4 million individuals. Lancet. 2018;391(10120):572-80.

7. Reeves D, Springate DA, Ashcroft DM, Ryan R, Doran T, Morris R, et al. Can analyses of electronic patient records be independently and externally validated? The effect of statins on the mortality of patients with ischaemic heart disease: a cohort study with nested case–control analysis. 2014;4(4):e004952.
8. Alvarez-Madrazo S, McTaggart S, Nangle C, Nicholson E, Bennie M. Data Resource Profile: The Scottish National Prescribing Information System (PIS). International Journal of Epidemiology. 2016;45(3):714-5f.
9. The Scottish Government. Scottish Index of Multiple Deprivation 2012. In: National Statistics, editor. Edinburgh2012.
10. Wickham H, Averick M, Bryan J, Chang W, McGowan LDA, François R, et al. Welcome to the Tidyverse. Journal of open source software. 2019;4(43):1686.
11. Therneau TM. A Package for Survival Analysis in R. R package version 3.3-1 https://CRAN.R-project.org/package=survival ed2022.
12. Kassambara A, Kosinski M, Biecek P. survminer: Drawing Survival Curves using `ggplot2'. R package version 0.4.9 https://CRAN.R-project.org/package=survminer ed2021.
13. Ripley B, Lapsley M. RODBC: ODBC Database Access. R package version 1.3-19 https://CRAN.R-project.org/package=RODBC ed2021.
14. Garnier S, Ross N, Rudis R, Camargo AP, Sciaini M, Scherer C. viridis - Colorblind-Friendly Colro Maps for R. R package version 0.6.2 https://sjmgarnier.github.io/viridis/ ed2021.
15. Gray R. cmprsk: Subdistribution Analysis of Competing Risks. R package version 2.2-10 https://CRAN.R-project.org/package=cmprsk ed2020.
16. Grolemund G, Wickham H. Dates and Times Made Easy with lubridate. Journal of Statistical Software. 2011;40(3):1--25.
17. Robinson D, Hayes A, Couch S. broom: Convert Statistical Objects into Tidy Tibbles. R package version 0.7.10 https://CRAN.R-project.org/package=broom ed2021.
18. Tang Y, Horikoshi M, Li W. ggfortify: unified interface to visualize statistical results of popular R packages. R J. 2016;8(2):474.
19. Wickham H. forcats: Tools for Working with Categorical Variables (Factors). R package version 0.5.1 https://CRAN.R-project.org/package=forcats ed2021.
20. Auguie B. gridExtra: Miscellaneious Functions for "Grid" Graphics. R package version 2.3 https://CRAN.R-project.org/package=gridExtra ed2017.
21. Kassambara A. ggpubr: `ggplot2' Based Publication Ready Plots. R package version 0.4.0 https://CRAN.R-project.org/package=ggpubr ed2020.
22. Wickham H. Reshaping Data with the reshape Package. Journal of Statistical Software. 2007;21(12):1--20.
23. National Records of Scotland. Mid-year population estimates by pre-April 2014 NHS Board areas by single year of age and sex: 1981-2013 Population Estimates Time Series Data: 'National Records of Scotland'; 2018.
24. Graham FJ, Friday JM, Pellicori P, Greenlaw N, Cleland JG. Assessment of haemoglobin and serum markers of iron deficiency in people with cardiovascular disease. Heart (British Cardiac Society) [Internet]. 2023 2023/08//; 109(17):[1294-301 pp.]. Available from: http://europepmc.org/abstract/MED/37130747

https://heart.bmj.com/content/heartjnl/early/2023/05/01/heartjnl-2022-322145.full.pdf

25. Lash TL, Fox MP, MacLehose RF, Maldonado G, McCandless LC, Greenland S. Good practices for quantitative bias analysis. International Journal of Epidemiology. 2014;43(6):1969-85.
